# *Pichia pastoris*-Expressed Bivalent Virus-Like Particulate Vaccine Induces Domain III-Focused Bivalent Neutralizing Antibodies without Antibody-Dependent Enhancement *in Vivo*

**DOI:** 10.3389/fmicb.2017.02644

**Published:** 2018-01-09

**Authors:** Rahul Shukla, Ravi K. Rajpoot, Upasana Arora, Ankur Poddar, Sathyamangalam Swaminathan, Navin Khanna

**Affiliations:** ^1^Recombinant Gene Products Group, Molecular Medicine Division, International Centre for Genetic Engineering and Biotechnology, New Delhi, India; ^2^Translational Health Science and Technology Institute, NCR Biotech Science Cluster, Faridabad, India; ^3^Department of Pediatrics, Emory University School of Medicine, Atlanta, GA, United States

**Keywords:** dengue virus, recombinant tetravalent vaccine, virus-like particle, envelope protein, antibodydependent enhancement, AG129 model, *Pichia pastoris*

## Abstract

Dengue, a significant public health problem in several countries around the world, is caused by four different serotypes of mosquito-borne dengue viruses (DENV-1, -2, -3, and -4). Antibodies to any one DENV serotype which can protect against homotypic re-infection, do not offer heterotypic cross-protection. In fact, cross-reactive antibodies may augment heterotypic DENV infection through antibody-dependent enhancement (ADE). A recently launched live attenuated vaccine (LAV) for dengue, which consists of a mixture of four chimeric yellow-fever/dengue vaccine viruses, may be linked to the induction of disease-enhancing antibodies. This is likely related to viral interference among the replicating viral strains, resulting in an unbalanced immune response, as well as to the fact that the LAV encodes prM, a DENV protein documented to elicit ADE-mediating antibodies. This makes it imperative to explore the feasibility of alternate ADE risk-free vaccine candidates. Our quest for a non-replicating vaccine centered on the DENV envelope (E) protein which mediates virus entry into the host cell and serves as an important target of the immune response. Serotype-specific neutralizing epitopes and the host receptor recognition function map to E domain III (EDIII). Recently, we found that *Pichia pastoris*-expressed DENV E protein, of all four serotypes, self-assembled into virus-like particles (VLPs) in the absence of prM. Significantly, these VLPs displayed EDIII and elicited EDIII-focused DENV-neutralizing antibodies in mice. We now report the creation and characterization of a novel non-replicating recombinant particulate vaccine candidate, produced by co-expressing the E proteins of DENV-1 and DENV-2 in *P. pastoris*. The two E proteins co-assembled into bivalent mosaic VLPs (mVLPs) designated as mE1E2_bv_ VLPs. The mVLP, which preserved the serotype-specific antigenic integrity of its two component proteins, elicited predominantly EDIII-focused homotypic virus-neutralizing antibodies in BALB/c mice, demonstrating its efficacy. In an *in vivo* ADE model, mE1E2_bv_ VLP-induced antibodies lacked discernible ADE potential, compared to the cross-reactive monoclonal antibody 4G2, as evidenced by significant reduction in the levels of IL-6 and TNF-α, suggesting inherent safety. The results obtained with these bivalent mVLPs suggest the feasibility of incorporating the E proteins of DENV-3 and DENV-4 to create a tetravalent mVLP vaccine.

## Introduction

Dengue is a mosquito-borne viral disease, prevalent in >100 tropical and sub-tropical countries, which poses a public health threat to ∼3.6 billion of the world population (Gubler, 2012). It is estimated to cause ∼390 million infections around the world annually, of which ∼25% result in clinical illness (Bhatt et al., 2013), which can range from mild self-limiting dengue fever or severe and potentially fatal dengue hemorrhagic fever and dengue shock syndrome (World Health Organization [WHO], 2017). Dengue infections can be caused by any one of four antigenically distinct serotypes of dengue viruses, DENV-1, DENV-2, DENV-3, or DENV-4, all of which belong to the family *Flaviviridae* (Lindenbach et al., 2013; Pierson and Diamond, 2013). Patients, who have experienced a primary infection with a particular DENV serotype, while being protected for life against re-infection by the same DENV serotype, tend to be predisposed to secondary infection by other DENV serotypes. Cross-reactive antibodies from prior exposure form immune complexes (ICs) with heterotypic DENVs, and mediate the latter’s increased uptake into Fc receptor (FcR)-bearing cells. This antibody-dependent enhancement (ADE) of secondary infection is widely held to be responsible for severe manifestations of dengue disease (Halstead, 2003). As partial immunity to dengue is associated with risk of ADE, a safe dengue vaccine must be tetravalent and confer simultaneous immunity to all four DENV serotypes.

A live attenuated vaccine (LAV) for dengue has become available recently (Ferguson et al., 2016; World Health Organization [WHO], 2017). This vaccine known as Dengvaxia is a mixture of four chimeric yellow fever virus (YFV)-based monovalent vaccines. Each monovalent viral vaccine is created by replacing two YFV structural genes, encoding the pre-membrane (prM), and the envelope (E) protein, with the *prM* and *E* genes of each of the four DENV serotypes (Swaminathan et al., 2010). Unfortunately, this vaccine manifested lower than anticipated efficacy, especially against DENV-2 in phase III trials (Capeding et al., 2014; Villar et al., 2015) and appeared to predispose younger children to increased risk of hospitalization (Hadinegoro et al., 2015). Preliminary analysis of these data suggests that Dengvaxia may be linked to ADE in younger vaccine recipients (Halstead and Russell, 2016). LAVs appear to be associated with viral interference which can compromise balanced tetravalent immunity (Edelman, 2011; Thomas, 2011; Swaminathan et al., 2013). This has underscored the need to find alternate non-replicating vaccine candidates that may eliminate or minimize ADE risk (Manoff et al., 2015; Vannice et al., 2015).

Virus-like particles (VLPs) offer an attractive non-replicating dengue vaccine alternative. It is well-known that DENV structural proteins prM and E, when co-expressed in heterologous hosts form non-infectious VLPs (Wang et al., 2009; Kuwahara and Konishi, 2010; Liu et al., 2010; Tang et al., 2012; Suphatrakul et al., 2015). From a vaccine perspective, the E protein offers the ideal dengue immunogen. It is a ∼500 amino acid (*aa*) residue long multi-functional glycoprotein, essential for host receptor recognition, membrane fusion and the major target of the host neutralizing immune response (Lindenbach et al., 2013; Pierson and Diamond, 2013). The amino-terminal 80% of the E protein, known as the ectodomain, is held together by six S–S bonds and is organized into discrete domains, envelope domain I (EDI), EDII, and EDIII (Modis et al., 2003). EDIII which mediates the host receptor recognition function of the E protein also contains multiple potent and type-specific virus-neutralizing epitopes (Gromowski and Barrett, 2007; Shrestha et al., 2010). On the other hand, the prM protein, which plays a role in virus maturation (Lindenbach et al., 2013), elicits ADE-causing antibodies (Dejnirattisai et al., 2010; Rodenhuis-Zybert et al., 2010; Smith et al., 2016). Thus, VLPs lacking prM are likely to be promising alternate dengue vaccine candidates.

We recently showed that the recombinant E ectodomain of each of DENV-1 (Poddar et al., 2016), DENV-2 (Mani et al., 2013), DENV-3 (Tripathi et al., 2015), and DENV-4 (Khetarpal et al., 2017), produced using the methylotrophic yeast *Pichia pastoris*, can self-assemble into immunogenic VLPs, in the absence of prM protein. These VLPs elicited predominantly serotype-specific neutralizing antibodies (nAbs) in mice. It is now feasible for us to envisage a tetravalent dengue vaccine candidate based on these monovalent E VLPs. However, we felt that while these monovalent E VLPs offer a promising vaccine candidate, the development of a tetravalent vaccine would entail the production of four monovalent vaccine VLPs. Instead, if we could co-express the E proteins of different DENV serotypes in a single yeast host, it may contribute to making the vaccine much more cost-effective, especially as dengue is endemic in the resource-poor regions of the globe. In a step in this direction, we sought to co-express and co-purify the DENV-1 E protein and the DENV-2 E protein, henceforth referred to as E1 and E2 proteins for convenience, and assess their vaccine potential. To this end, we sought to address the following questions: Can E1 and E2 be efficiently co-expressed in a single *P. pastoris* host? Would these two proteins co-assemble to form mosaic VLPs (mVLPs)? If they did, would these mVLPs preserve the antigenic integrity of E1 and E2 epitopes? If so, would these mVLPs elicit immune responses specific to DENV-1 and DENV-2? Would they manifest ADE potential? We present data supporting the feasibility of generating such bivalent mVLPs, which preserve the antigenic integrity of its monovalent constituents and capable of eliciting nAbs to DENV serotypes 1 and 2. Using a recently reported murine ADE model (Watanabe et al., 2015) which manifests increased vascular permeability accompanied by the production of elevated levels of pro-inflammatory cytokines, tumor necrosis factor-α (TNF-α), and interleukin-6 (IL-6), we also present data demonstrating that these mVLPs may be inherently safe as they appear to lack ADE potential.

## Materials and Methods

### Ethics Statement

All animal experiments were performed in compliance with the animal ethical guidelines of the Committee for the Purpose of Control and Supervision of Experiments on Animals (CPCSEA) of Government of India. Experimental protocols involving the use of BALB/c (ICGEB/IAEC/08/2016/RGP-14) and AG129 (ICGEB/IAEC/08/2016/RGP-15) mice were approved by the Institutional Animal Ethics Committees (IAEC) of International Centre for Genetic Engineering and Biotechnology, New Delhi.

### DENV *E* Genes, Cells, Viruses, Antibodies, and Other Reagents

The *E1* (*DENV-1 E*, GenBank accession no: JX292264) and *E2* (*DENV-2 E*, GenBank accession no: JX292265) genes, codon-optimized for *P. pastoris* expression have been described before (Mani et al., 2013; Poddar et al., 2016). *E. coli* DH5α, *P. pastoris* strain GS115 and the yeast integrative plasmid pAO815 were from Invitrogen (Thermo Fisher Scientific), Carlsbad, CA, United States. C6/36 and Vero cells were obtained from American Type Culture Collection (ATCC), Manassas, VA, United States. C6/36 cells were maintained at 28°C in Leibovitz L-15 medium, containing 0.03% (vol/vol) tryptose phosphate broth and 10% (vol/vol) heat-inactivated fetal bovine serum (ΔFBS), in a CO_2_-free incubator. Vero cells were maintained in Dulbecco’s Modified Eagle Medium supplemented with 10% ΔFBS, in 10% CO_2_ humidified incubator. The WHO reference strains of the four DENV serotypes [DENV-1 (WP 74), DENV-2 (S16803), DENV-3 (CH53489), and DENV-4 (TVP-360)] have been reported earlier (Kraus et al., 2007). DENV stocks, prepared by infecting C6/36 cells, were titrated on Vero cells using fluorescence activated cell sorting (FACS) assay. Viral titers were expressed as FACS infectious units (FIU)/ml, calculated using the formula: FIU/ml = [(% infected cells) × (total number of cells in well) × (dilution factor)]/(volume of virus inoculum), as described (Lambeth et al., 2005). The DENVs and *E. coli* clones expressing Maltose Binding Protein (MBP), MBP-EDIII-1 (MBP fused in frame to EDIII domain of DENV-1), MBP-EDIII-2, MBP-EDIII-3, and MBP-EDIII-4 fusion proteins were kind gifts from Dr. Aravinda de Silva, University of North Carolina (UNC), United States. All these *E. coli*-expressed proteins were purified in-house. *P. pastoris*-expressed recombinant E proteins corresponding to the four DENV serotypes (Mani et al., 2013; Tripathi et al., 2015; Poddar et al., 2016; Khetarpal et al., 2017) as well as all type-specific and cross-reactive murine and human monoclonal antibodies (mAbs) used in this study have been described before (Henchal et al., 1982; Brien et al., 2010; Shrestha et al., 2010; Sukupolvi-Petty et al., 2010, 2013; Wahala et al., 2010; De Alwis et al., 2011, 2014; Smith et al., 2012, 2013). Anti-mouse immunoglobulin G (IgG)-horse radish peroxidase (HRPO) conjugate for ELISA was purchased from Calbiochem, San Diego, CA, United States. The HRPO substrate 3, 3′, 5, 5′-Tetramethylbenzidine (TMB), concanavalin A (Con A)-HRPO conjugate and acid-washed glass beads were procured from Sigma–Aldrich (St. Louis, MO, United States). Zymolyase was purchased from G-Biosciences (St. Louis, MO, United States). Ni-NTA resin and RNeasy mini kit were purchased from Qiagen (Hilden, Germany). High binding polystyrene ELISA plates were from Corning Incorporated (Corning, NY, United States).

### Generation of a *P. pastoris* Clone Capable of Simultaneous Expression of the *E* Genes of DENV-1 and DENV-2

The pPICZA-based plasmids expressing the *E* genes of DENV-1 (Poddar et al., 2016) and DENV-2 (Mani et al., 2013), encoding the prM signal peptide followed by the cognate E ectodomain plus 6x His tag, described earlier, were used as templates for PCR-based introduction of *Eco* RI sites at either ends of the *E1* and *E2* genes, to facilitate cloning of each of the amplified inserts, separately, into the unique *Eco* RI site of pAO815. This resulted in the creation of two monovalent plasmids, one containing the *E1* gene (pDENV-E1) and the other containing the *E2* gene (pDENV-E2), both of which were sequence-verified. In these monovalent plasmids, the *E* gene is under the transcriptional control of the alcohol oxidase 1 (*AOX1*) promoter. The *E2* expression cassette (EC), the fragment containing the *AOX1* promoter-*E2* gene-*AOX1* transcriptional terminator, was recovered from pDENV-E2 by digestion with *Bam* HI and *Bgl* II and inserted into the unique *Bam* HI site of pDENV-E1, located at the 3′ side of the *E1* EC, to obtain the bivalent plasmid pDENV-E1E2_bv_. This plasmid, verified by restriction analysis, was digested with *Bgl* II to obtain a linear DNA fragment containing the *E1* and *E2* ECs plus the *HIS4* marker, which was integrated into the *AOX1* locus of *P. pastoris* GS115 (*his4*) by electroporation according to the vendor’s protocol. Transformants were selected on minimal plates lacking histidine and analyzed further for slow methanol utilization (*mut*^S^) phenotype. His^+^/*Mut*^S^ clones were analyzed for genomic integration of both *E1* and *E2* genes by extracting their genomic DNA and subjecting them to PCR using primer pairs specific to *E1* (FP1 and RP1) and *E2* (FP2 and RP2) genes which were designed to generate two unique amplified products from the two genes.

### Analysis of Co-expression of Both Integrated Genes *E1* and *E2*

Methanol-induced bivalent *P. pastoris* cells were washed with 1× phosphate buffered saline (PBS) and re-suspended in sterile water containing 0.3 M DTT (∼10^7^ cells in 70 μl) and incubated with 1.5 units zymolyase at 37°C for 2 h to lyse the cells. Total RNA was isolated from the resultant lysate using RNeasy mini kit. This RNA (5 μl) was reverse transcribed (20 μl reaction volume) either with RP1 (complementary to *E1* mRNA) or RP2 (complementary to *E2* mRNA) primers using Bio-Rad’s iScript reverse transcriptase (42°C/1 h, to synthesize cDNA, followed by 85°C/5 min to inactivate the reverse transcriptase). The resulting cDNA (2 μl of a 10-fold dilution) was subjected to real-time PCR (50 μl reaction volume) in the presence of both the cognate forward and reverse primers (FP1 and RP1 for *E1* cDNA; FP2 and RP2 for *E2* cDNA) using iQ SYBR green Supermix, in a Bio-Rad MiniOpticon real-time PCR machine (cycling parameters: 94°C/4 min × 1 cycle; 94°C/40 s; 61°C/30 s; 72°C/1 min × 42 cycles; melt curve analysis: 55–95°C). For comparison total RNA extracted from equivalent number of cells of methanol-induced monovalent *P. pastoris* clones harboring the *E1* gene (Poddar et al., 2016) and the *E2* gene (Mani et al., 2013) were also subjected to reverse transcription (RT)-qPCR, in parallel.

### Methanol-Induced Expression

Cultures of the selected bivalent *P. pastoris* clone harboring the two ECs were grown to log phase in yeast extract-peptone-dextrose medium and induced using methanol. To identify optimal methanol concentration and induction duration, both parameters were varied individually, keeping the other fixed. E1 and E2 protein expression was detected in ELISAs and Western blots using mAb 24A12 (Batra et al., 2010) which is specific to the EDIIIs of both these proteins. This analysis revealed that methanol at 1.25% (v/v) added at 12-hourly intervals for 72 h is required to achieve maximal induction.

### Localization of E1 and E2 Protein Expression

An aliquot of methanol-induced culture (equivalent to 100 OD_600_) of the bivalent clone was lysed with glass beads (450 microns) in 0.5 ml of lysis buffer (50 mM Tris-HCl buffer, pH 8.5/500 mM NaCl) and separated into supernatant (S) and membrane-enriched pellet (P) fractions. The latter was solubilized in lysis buffer containing 8 M urea and clarified by centrifugation. The E1 and E2 proteins in the ‘S’ and ‘P’ fractions were detected using Ni^2+^-NTA His-Sorb ELISA in conjunction with mAb 24A12 as before (Batra et al., 2010). For comparison, methanol-induced lysates prepared from the monovalent *E1* and *E2* gene-expressing *P. pastoris* clones were also separated into ‘S’ and ‘P’ fractions and analyzed using Ni^2+^-NTA His-Sorb ELISA, in parallel.

### Purification and Characterization of E1 and E2 Proteins Expressed by the Bivalent Clone

Purification was performed using Ni^2+^-NTA affinity chromatography under denaturing conditions starting from ∼100 g induced biomass, essentially as reported before (Mani et al., 2013). Purified proteins were characterized by SDS–PAGE and Western blot using mAb 24A12 in conjunction with anti-mouse IgG-HRPO conjugate. Glycosylation status of the protein was analyzed in protein blots using Con A-HRPO. Both Western and protein blots were developed using TMB substrate.

### Evaluation of Mosaic VLP Formation

The presence of both E1 and E2 proteins in the purified material was ascertained using mAbs specific to each of these two proteins in a customized time-resolved fluorescence (TRF)-based sandwich assay. This assay was a modification of a previously designed TRF assay in a sandwich format using Europium III (Eu^3+^) as a tracer (Talha et al., 2011). Briefly, low-fluorescence microtiter wells were coated with a DENV-1-specific mAb, washed and treated with an aliquot of the affinity purified material. Wells were washed once again and incubated with DENV-2-specific mAb 3H5 conjugated to Eu^3+^ chelate (Talha et al., 2011). TRF of the bound tracer was measured using Victor^3^V 1420 Multiplate counter (λ_ex_ 340 nm; λ_em_ 615 nm). Assembly into higher order particulate forms in the affinity purified material (adjusted to 200 μg protein content/ml of 20 mM Tris-HCl containing 50 mM NaCl, pH 8.5) was evaluated by dynamic light scattering (DLS) using Zetasizer Nano ZS90 (Malvern Instruments, Malvern, United Kingdom). To visualize VLP formation, the purified material (adjusted to 5–10 μg protein content/ml of 1× PBS, pH 7.2) was applied onto carbon-coated EM grid (1–2 min), negatively stained with 1% uranyl acetate (30 s), washed with Milli-Q water and examined using a Tecnai electron microscope (EM).

### Analysis of DENV-1 and DENV-2 Type-Specific Epitope Integrity of the mE1E2_bv_ VLPs

Indirect ELISA was performed to examine the antigenic integrity of the mE1E2_bv_ VLPs purified from the methanol-induced bivalent *P. pastoris* clone. To this end, the affinity-purified mE1E2_bv_ VLPs were used to the coat the ELISA plate mictotiter wells to determine the reactivity toward a panel of human and murine type-specific and cross-reactive DENV mAbs (Henchal et al., 1982; Brien et al., 2010; Shrestha et al., 2010; Sukupolvi-Petty et al., 2010, 2013; Wahala et al., 2010; De Alwis et al., 2011, 2014; Smith et al., 2012, 2013). Bound antibodies were revealed using the cognate anti-human or anti-mouse secondary antibody-HRPO conjugate together with TMB substrate.

### Antigen Formulation

The mE1E2_bv_ VLPs (40 μg) were incubated with alhydrogel (500 μg) for 36 h at 4°C on a rocking platform. The suspension was centrifuged (5,000 rpm, 5 min) and the alhydrogel-bound antigen was re-suspended in 100 μl sterile 1× PBS. This represented one dose for immunization. This dose for the bivalent VLPs was based on previous work which showed that 20 μg monovalent DENV-2 E VLPs resulted in maximal immune response (Mani et al., 2013). The pmE1+E2 VLP formulation was prepared similarly, with the exception that equal amounts of the alhydrogel-bound E1 and E2 antigens were re-suspended in 100 μl sterile 1× PBS such that a single dose comprised 20 μg each of the two monovalent VLPs coated onto 500 μg alhydrogel. Mock formulation contained 500 μg alhydrogel/100 μl sterile 1× PBS per dose.

### Mouse Immunization

A group of 4–6 week old BALB/c mice (*n* = 10) were immunized intraperitoneally with 40 μg mE1E2_bv_ VLPs coated on 500 μg alhydrogel (in 100 μl 1× PBS) per dose using a three dose schedule (0, 30, and 90 days) as reported earlier (Mani et al., 2013; Tripathi et al., 2015; Poddar et al., 2016; Khetarpal et al., 2017). For comparison, we also formulated a physical mixture of monovalent E1 (Poddar et al., 2016) and E2 (Mani et al., 2013) VLPs (20 μg each coated on alhydrogel), designated as pmE1+E2 VLPs, and immunized a second group of 10 mice. A third group was mock-immunized with PBS + alhydrogel. Mice were bled retro-orbitally on day 97 for seroanalysis.

### Seroanalysis

Serial dilutions of the pooled anti-mE1E2_bv_ VLP antisera were analyzed for antibodies to three sets of coating antigens by indirect ELISA. The coating antigens used were: (i) the four recombinant envelope proteins E1, E2, E3, and E4, (ii) the four recombinant MBP-EDIII fusion proteins, MBP-EDIII-1, MBP-EDIII-2, MBP-EDIII-3, and MBP-EDIII-4, and (iii) the four viruses, DENV-1, DENV-2, DENV-3, and DENV-4. Bound antibodies were detected using anti-mouse IgG-HRPO conjugate plus TMB substrate.

Virus-nAb titers in serially diluted murine sera, against a WHO panel of DENVs (Kraus et al., 2007), were determined using FACS-based assay as before (Mani et al., 2013; Tripathi et al., 2015; Poddar et al., 2016; Khetarpal et al., 2017). The FACS neutralization titer (FNT_50_) is the serum dilution capable of causing a 50% decrease in the number of DENV-infected cells compared to the corresponding control infection performed in the absence of any immune serum. In some experiments, the murine immune serum was selectively depleted (see below) of EDIII-specific antibodies by pre-incubation with amylose resin bound to MBP fused in-frame to either EDIII-1 or EDIII-2. Control depletion of an equivalent aliquot of immune serum was done using MBP alone bound to amylose resin. Residual virus-nAb titers in the depleted sera were determined using the FACS-based assay as before.

### Antibody Depletion of Antisera

Antisera were subjected to depletion of EDIII-specific antibodies as follows. Aliquots of amylose resin (100 μl), washed thoroughly (in 20 mM Tris-HCl/200 mM NaCl/1 mM EDTA, pH 7.4) were separately incubated overnight at 4°C with purified MBP, MBP-EDIII-1, or MBP-EDIII-2 (0.3 mg each) on a rocking platform. Resin containing bound protein was washed (with wash buffer, followed by 1× PBS, three times each) and blocked with 1% bovine serum albumin (prepared in 1× PBS) for 2 h at 37°C. The blocked resin was washed with 1× PBS (3 times) and incubated with 1 ml of 1:10 diluted antiserum for 45 min at 37°C. The depleted serum was recovered by centrifugation (3,000 rpm/5 min/room temperature) and used for FACS-based virus neutralization assay

### Evaluation of ADE Potential of Antibodies Elicited by mE1E2_bv_ VLPs

The ADE potential of the anti-mE1E2_bv_ VLP antibodies was evaluated using a recently developed method (Watanabe et al., 2015) in the AG129 mouse model (Balsitis et al., 2010; Zellweger et al., 2010). ICs were generated *in vitro* by incubating aliquots of the challenge strain DENV-2 S221 (2 × 10^4^ FIU) separately with adequate amounts of mAb 4G2 (10 μg), anti-mE1E2_bv_ VLP antiserum (5 μl neat), anti-mE3E4_bv_ VLP antiserum (5 μl neat), or anti-DENV-2 S221 antiserum (5 μl neat) for 60 min on ice (in 50 μl volume). Each of these ICs (ascertained to be fully neutralized in the FACS assay) was prepared in amounts adequate for injecting (intravenously) into nine AG129 mice (50 μl IC/mouse). Two control groups were also set up with one group receiving mAb 4G2 (10 μg/mouse in 50 μl) alone and the other receiving DENV-2 S221 (2 × 10^4^ FIU in 50 μl volume) alone. All groups were monitored for survival for the indicated duration and the data used to generate Kaplan–Meier survival curves.

### Cytokine Analysis

Three mice from each of the groups in the ADE experiment above were euthanized on day 3 after IC inoculation for collecting tissue samples. After extensive perfusion with 1× PBS, and removal of all visible luminal content, the small intestines were collected and snap frozen in liquid nitrogen. Tissue extracts were prepared in 1× PBS using a Polytron homogenizer and clarified by centrifugation. TNF-α and IL-6 in the clarified small intestinal extracts were determined using commercial cytokine ELISA kits (cat# KMC3011 for TNF-α; cat# KMC0061 for IL-6) procured from Invitrogen as per the manufacturer’s instructions. Cytokine concentrations were determined with reference to recombinant biotinylated murine TNF-α and IL-6 standards provided in the kits.

### Statistical Analyses

Statistical significance of the difference between data sets was determined using two-tailed Student’s *t*-test. Statistical calculations were performed using GraphPad Prism software. Kaplan–Meier survival curves were analyzed by the Mantel–Cox test for significance. Probability (*p*) < 0.05 was considered statistically significant.

## Results

### Strategy to Co-express *E* Proteins of DENV-1 and DENV-2 in *P. pastoris*

E1 and E2 monovalent ECs were created first by inserting codon-optimized synthetic genes *E1* and *E2*, respectively, into the unique *Eco* RI site of the *P. pastoris* integrative vector pAO815 (Supplementary Figure S1). Each of these genes encodes the C-terminal 34 *aa* residues of prM protein, followed by the first 395 *aa* residues of the E protein of the cognate DENV serotype and a 6× His tag (Mani et al., 2013; Poddar et al., 2016). To achieve co-expression of both E1 and E2 proteins using a single plasmid vector, the E2 EC (retrieved from pDENV-E2) was inserted into pDENV-E1 on the 3′ side of the E1 EC (Supplementary Figure S2). The resultant construct carries the E1 and E2 protein ECs in a head-to-tail tandem arrangement. A map of this bivalent expression construct, pDENV-E1E2_bv_, is shown in Supplementary Figure S2. It was verified by restriction and PCR analyses (Supplementary Figure S3). The two ECs of the bivalent plasmid together with the adjoining *HIS4* marker were integrated into the *AOX1* locus of the His^-^ auxotrophic *P. pastoris* strain GS115 (*his4*).

**FIGURE 1:**
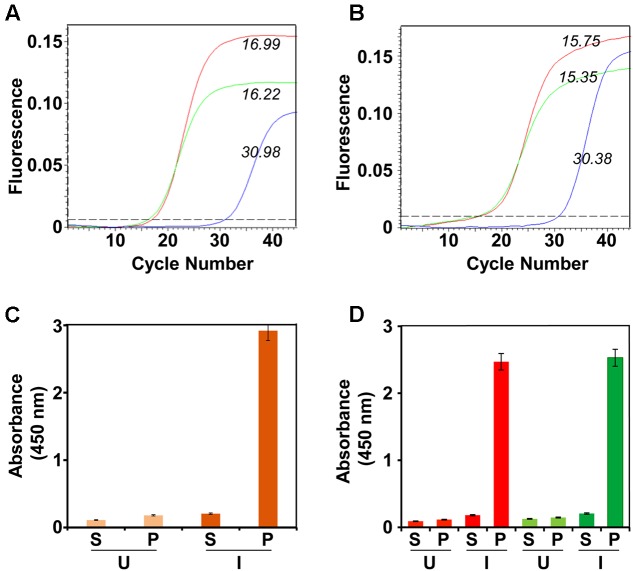
Expression and localization of DENV E1E2_bv_. **(A)** Real time analysis of DENV *E1* and *E2* mRNA expression in *Pichia pastoris* transformed with pDENV-E1E2_bv_. Total RNA was isolated from the methanol-induced bivalent clone of *P. pastoris*, transcribed to cDNA using reverse primers specific to either *E1* (red) or *E2* (green) and subjected to qPCR. Total RNA from *P. pastoris* transformed with the empty expression vector pAO815 was subjected to RT-qPCR in parallel as a negative control (blue). **(B)** RT-qPCR amplification profiles obtained using total RNA extracted from un-transformed *P. pastoris* host (blue) or monovalent *P. pastoris* clones expressing E1 (red) or E2 (green). In **(A,B)**, the italicized Arabic numerals indicate *C*_t_ values. **(C)** Localization of E1E2_bv_ expression. The bivalent *P. pastoris* clone (orange bars) lysed either before (U) or after (I) methanol induction and separated into the supernatant (S) and pellet (P) fractions was analyzed by His-Sorb ELISA using a dengue specific mAb 24A12. **(D)** Similar experiment, as in ‘**C**,’ performed in parallel with monovalent *P. pastoris* clones expressing E1 (red bars) or E2 (green bars) proteins.

We next analyzed the co-expression of the *E1* and *E2* genes in the selected bivalent clone after methanol induction (**Figure 1A**). Total RNA was isolated from the induced cells of the bivalent clone and subjected to RT followed by q-PCR using primers specific to *E1* and *E2* genes (Supplementary Table S1). This revealed that the induced bivalent cells expressed mRNAs corresponding to the two different DENV *E* genes. Further, the *C*_t_ values suggested that both genes are expressed efficiently. In parallel, we also performed RT-qPCR analysis using total RNA extracted from the previously reported monovalent *P. pastoris* clones harboring the *E1* (Poddar et al., 2016) and *E2* (Mani et al., 2013) genes. We used identical induction conditions for the monovalent clones and took similar number of induced cells (based on OD_600_) for RNA extraction. This analysis (**Figure 1B**) revealed that the amplification profiles and *C*_t_ values of the two monovalent clone-expressed mRNAs were very similar and consistent to those observed for the two *E* genes expressed by the bivalent clone. This suggests that the bivalent clone expresses each of the two DENV *E* genes as efficiently as do the corresponding monovalent clones. Using a Western blot approach we have shown before that monovalent E1 (Poddar et al., 2016) and E2 (Mani et al., 2013) proteins are predominantly associated with the membrane-enriched pellet fraction of induced cells lysed in non-denaturing buffers. Using His-Sorb ELISA in conjunction with mAb 24A12 (Batra et al., 2010), an ‘in house’ mAb, which recognizes the E proteins of DENV serotypes 1 and 2 (as well as serotype 3), we found that the bivalent clone-expressed proteins (designated E1E2_bv_) are associated with the pellet fraction of the native lysate (**Figure 1C**). Using this assay, the E1 and E2 proteins expressed by the monovalent clones were also found to be associated with the pellet fraction (**Figure 1D**), consistent with previous observations (Mani et al., 2013; Poddar et al., 2016).

### Co-purification of E1 and E2

**FIGURE 2:**
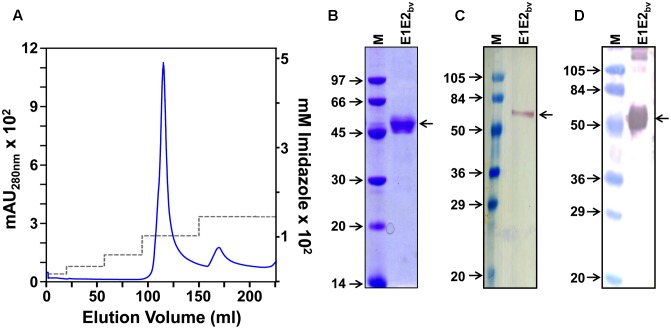
Purification and preliminary characterization of recombinant E1E2_bv_. **(A)** Ni^2+^-NTA affinity chromatographic purification of E1E2_bv_. The solid blue curve represents the UV absorbance at 280 nm. The imidazole step gradient is shown by the dashed gray line. **(B)** SDS–PAGE analysis of the purified material (pooled peak fractions from ‘**A**’) visualized by Coomassie blue staining. **(C)** Western blot of the purified preparation using mAb 24A12 in conjunction with anti-mouse IgG-HRPO. **(D)** Protein blot showing the interaction of the purified material with Con A-HRPO. Low molecular weight protein markers **(B)** or pre-stained protein markers **(C,D)** were run in lanes marked ‘M’; their sizes (kDa) are shown to the left of each panel. The position of E1E2_bv_ is shown by the arrow on the right side of **(B–D)**.

The membrane-enriched pellet fraction from a native lysate of the induced bivalent clone biomass was solubilized using a denaturing buffer and chromatographed on a Ni^2+^-NTA column under denaturing conditions. After washing away unbound/non-specifically bound material, bound proteins were eluted with an imidazole step gradient. A single major protein peak emerged from the column at 100 mM imidazole as depicted in **Figure 2A**. Column fractions across this peak were pooled and subjected to SDS–PAGE followed by Coomassie staining to reveal a single major protein band of ∼45 kDa size (**Figure 2B**). That this band indeed contains the DENV E proteins was corroborated by immunoblotting with mAb 24A12 which recognize both E1 and E2 (**Figure 2C**). As the RT-qPCR data revealed that both *E1* and *E2* mRNAs are co-expressed in the bivalent clone, we assumed that the major protein band in the pooled peak material contains E1 and E2 proteins (as demonstrated experimentally below), both of which, being similar in molecular size, co-migrate together as a single band on SDS–PAGE. Based on densitometric analysis the purity was judged to be >95%. Our earlier studies had shown that the monovalent E proteins expressed by *P. pastoris* are glycosylated (Mani et al., 2013; Tripathi et al., 2015; Poddar et al., 2016; Khetarpal et al., 2017). Consistent with this, a protein blot showed that the E proteins expressed by the bivalent *P. pastoris* clone are glycosylated, as evidenced by their interaction with Con A (**Figure 2D**). Based on multiple purifications, we obtain an average of ∼30–35 mg of >95% purified protein per L of induced culture.

### E1 and E2 Proteins Co-associate to Form Bivalent Mosaic VLPs Which Retain the Antigenic Integrity of Their Monovalent Precursors

**FIGURE 3:**
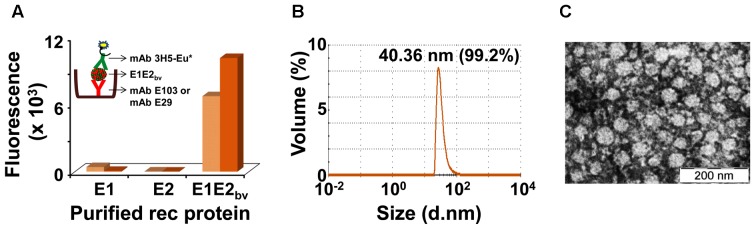
Additional physical characterization of E1E2_bv_. **(A)** Sandwich ELISA demonstrating the presence of E1 and E2 proteins in the purified material. The purified material was captured using DENV-1 mAb (mAb E103: light brown bars; mAb E29: dark brown bars) and revealed using DENV-2 mAb 3H5-Eu^∗^ conjugate. Inset depicts a schematic representation of the sandwich ELISA of mE1E2_bv_ VLPs. **(B)** DLS analysis of purified mE1E2_bv_ VLPs. The average diameter of the VLPs is shown at the top of the peak; the value in parenthesis indicates the proportion (in %) of these VLPs in the purified preparation. **(C)** EM picture of the VLPs after negative staining with uranyl acetate (the scale is shown on the bottom right).

As indicated above, it was expected that two proteins, E1 and E2, are present in the purified E1E2_bv_ preparation based on RT-qPCR data. Our earlier studies showed that both E1 and E2 proteins expressed by the monovalent *P. pastoris* clones have the capacity to self-assemble into VLPs (Mani et al., 2013; Poddar et al., 2016). This led us to speculate if the E1 and E2 proteins co-expressed by the bivalent clone may co-associate to form mVLPs. To test this, we devised a novel sandwich assay. In this assay, microtiter wells were coated with one of two different E1 protein-specific mAbs, E29 or E103 (Shrestha et al., 2010). The purified E1E2_bv_ protein preparation was applied onto these wells to capture the putative mVLPs through their E1 component. Following this, we used the E2-specific mAb 3H5 (Henchal et al., 1982), labeled with Eu^3+^ chelate (Talha et al., 2011) as a tracer, to detect the bound VLPs (**Figure 3A** inset). For comparison, we also tested purified monovalent E1 (Poddar et al., 2016) and E2 (Mani et al., 2013) VLPs in this sandwich assay. The results of this analysis, summarized in the bar diagram in **Figure 3A** revealed that the purified material obtained from the bivalent clone could be picked up in this sandwich assay designed to generate a signal only when both E1 and E2 are present simultaneously, presumably as mVLPs. Consistent with this, monovalent E1 and E2 VLPs did not result in any discernible fluorescence read-out in this assay.

While the sandwich assay proves that both E1 and E2 proteins interact with each other in the material purified from the bivalent clone, it does not provide any evidence of co-assembly into higher order structures. We used DLS to evaluate the presence of higher order structures in the purified E1E2_bv_ preparation from the bivalent clone. The DLS data, presented in **Figure 3B**, demonstrated that there were indeed particles of ∼40 nm comprising >99% of the material purified from the bivalent clone. Consistent with this, EM analysis revealed the presence of discrete VLPs in the purified material (**Figure 3C**). Taken together with the sandwich assay data, the DLS and EM data show that co-expression of E1 and E2 in a single host can lead to their co-assembly into mVLPs containing both co-expressed proteins. As these mVLPs contain E1 and E2 proteins, co-expressed by the bivalent clone, these are henceforth referred to as mE1E2_bv_ VLPs. In its current format, the sandwich ELISA, *per se*, is designed to detect the bivalent mVLPs, not the efficiency of the co-assembly process. However, our experience with EDIII-based mVLPs suggests the efficiency of co-assembly could be as high as 80–90%.

As mentioned earlier, EDIII possesses potent type-specific neutralizing epitopes (Gromowski and Barrett, 2007; Shrestha et al., 2010). Our previous studies with recombinant monovalent E proteins produced using *P. pastoris*, showed that the VLPs they form display EDIII efficiently on the surface (Mani et al., 2013; Tripathi et al., 2015; Poddar et al., 2016; Khetarpal et al., 2017). The question we addressed next was, do these mE1E2_bv_ VLPs preserve the antigenic integrity of the EDIIIs of the component E1 and E2 proteins? To this end, we probed these VLPs using a panel of DENV type- and complex-specific murine and human mAbs in indirect ELISAs (**Table 1**). Several mAbs such as E12, E24, E29, E37, and E103 (Shrestha et al., 2010), which are specific to EDIII of the E1 protein, manifested ELISA reactivity toward the mE1E2_bv_ VLPs as efficiently as they did toward monovalent E1 VLPs, while at the same time being virtually unreactive toward monovalent E2, E3, and E4 VLPs. This was essentially mirrored by the observed reactivity of the EDIII-2-specific mAbs 3H5 (Henchal et al., 1982), and 106, 104, and 70 (Sukupolvi-Petty et al., 2010), which recognized both mE1E2_bv_ VLPs and monovalent E2 VLPs with comparable efficiency, while at the same time manifesting no discernible ELISA reactivity toward monovalent E1, E3, and E4 VLPs. As expected, DENV-3 and DENV-4 EDIII-specific mAbs 8A1 (Wahala et al., 2010) and E88 (Sukupolvi-Petty et al., 2013), which recognized monovalent E3 and E4 VLPs, respectively, did not recognize the mE1E2_bv_ VLPs, as well as the monovalent E1 and E2 VLPs. That the mE1E2_bv_ VLPs also display EDIII on their surface as do the monovalent VLPs was corroborated by the reactivity of several DENV complex-specific murine mAbs E77 (Brien et al., 2010) and 12C1.5 (Wahala et al., 2010) as well as the human mAb 2J20 (Smith et al., 2012). It was also seen that the mE1E2_bv_ VLPs manifested reactivity toward cross-reactive murine (Henchal et al., 1982) and human (De Alwis et al., 2011; Smith et al., 2013) mAbs specific to EDI/II and FL. Consistent with design, the mE1E2_bv_ VLPs did not display any reactivity toward prM-specific mAb 2K2 (De Alwis et al., 2014). Overall, the mAb reactivity analysis leads to the conclusion that the mE1E2_bv_ VLPs preserve the antigenic integrity of the constituent E proteins, especially EDIII which is implicated in host cell receptor recognition and in the induction of potent nAbs.

**Table 1:** Analysis of E-specific epitope integrity of the mE1E2_bv_ VLPs.

mAb^a^	Serotype specificity	Epitope specificity^b^	ELISA OD_450__nm_^c^				
			mE1E2_bv_ VLPs	E1	E2	E3	E4
E103	DENV-1	EDIII LR	3.48	3.46	0.06	0.03	0.09
E24		EDIII	0.77	1.23	0.03	0.02	0.03
E37		EDIII	0.93	1.25	0.02	0.02	0.02
E29		EDIII	1.67	1.87	0.03	0.09	0.14
E12		EDIII	1.54	1.82	0.07	0.12	0.07

3H5	DENV-2	EDIII LR	3.45	0.14	3.46	0.06	0.11
106		EDIII LR	2.51	0.05	2.5	0.03	0.03
104		EDIII C-C LOOP	3.44	0.08	3.45	0.1	0.05
70		EDIII AS/LR	0.91	0.02	0.92	0.03	0.03

8A1	DENV-3	EDIII LR	0.06	0.04	0.07	1.39	0.05

E88	DENV-4	EDIII LR	0.08	0.06	0.04	0.07	2.93

E77	All four DENV serotypes	EDIII AS/LR	1.25	3.50	1.21	1.64	1.21
12C1.5		EDIII	3.76	3.66	3.86	3.21	3.01
4G2		FL	0.13	0.23	0.20	0.21	0.07
h-2J20		EDIII	3.03	3.67	3.46	1.67	1.08
h-3H4		EDII/III	1.13	0.11	0.16	0.15	0.12
h-1M7		FL	2.45	2.97	3.03	1.28	1.93
h-1N5		FL	0.37	0.95	0.45	0.19	0.13
h-DVC23.13		EDI/II	0.41	0.22	0.09	0.13	0.09
h-2K2		prM	0.01	0.02	0.02	0.01	0.02

^a^*mAbs with ‘h’ prefix are of human origin; the rest are murine mAbs; these mAbs are described in the following references: Shrestha et al. (2010) (mAbs E12, E24, E29, E37 and E103); Henchal et al. (1982) (mAbs 3H5 and 4G2); Sukupolvi-Petty et al. (2010) (mAbs 106, 104, 70); Sukupolvi-Petty et al. (2013) (mAb E88); Wahala et al. (2010) (mAbs 8A1 and 12C1.5); Brien et al. (2010) (mAb E77); Smith et al. (2012) (mAbs 2J20 and 3H4); Smith et al. (2013) (mAbs 1M7 and 1N5); De Alwis et al. (2011) (mAb DVC23.13); De Alwis et al. (2014) (mAb 2K2).*

^b^*Abbreviations are as follows: LR, lateral ridge; AS, A strand; FL, fusion loop.*

^c^*Data shown are the average of duplicate experiments*.

### mE1E2_bv_ VLPs Elicit Cross-Reactive Antibodies Skewed More toward DENV Serotypes 1 and 2

**FIGURE 4:**
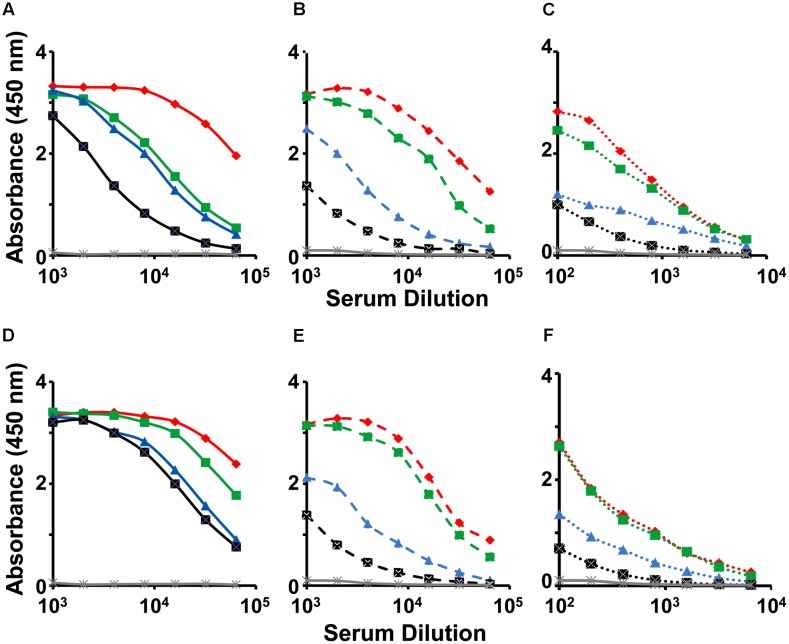
Evaluation of immunogenicity of mE1E2_bv_ VLPs. Antisera from BALB/c mice immunized with bivalent mE1E2_bv_ VLPs **(A–C)** or a physical mixture of monovalent VLPs (pmE1+E2 VLPs), corresponding to DENV serotypes 1 and 2 **(D–F)**, were analyzed by indirect ELISA using all four DENV-E **(A,D)** and all four EDIII **(B,E)** proteins and all four DENV **(C,F)** serotypes, as coating antigens. In each of the panels, DENV serotypes 1, 2, 3, and 4, are indicated in red, green, blue, and black, respectively. Mock-immunized (PBS on alhydrogel) BALB/c serum is represented in gray line in all the six panels. Data points are averages of duplicates.

We investigated the immunogenicity of the mE1E2_bv_ VLPs, formulated in alhydrogel, in BALB/c mice. For comparison we also tested the immunogenicity of a physical mixture of monovalent E1 and E2 VLPs (pmE1+E2 VLPs). Pooled antisera collected a week after administration of the three-dose regimen were first tested by indirect ELISA using three sets of four coating antigens each, namely, the recombinant E proteins (E1, E2, E3, and E4 proteins), the recombinant EDIII fusion proteins (MBP-EDIII-1, MBP-EDIII-2, MBP-EDIII-3, and MBP-EDIII-4), and the infectious DENVs (DENV-1, DENV-2, DENV-3, and DENV-4), corresponding to the four serotypes (**Figure 4**). The anti-mE1E2_bv_ VLP antisera manifested reactivity with purified recombinant E proteins of all four DENV serotypes (**Figure 4A**). This is consistent with the ∼60% sequence homology that exists among the E proteins of the four DENV serotypes. However, the reactivity toward E proteins of serotypes 1 and 2 were relatively higher compared to that of the remaining two serotypes. This was also mirrored by the relatively greater reactivity of the anti-pmE1+E2 antisera toward DENV serotypes 1 and 2, compared to serotypes 3 and 4 (**Figure 4D**). The preferential serotype-specific reactivity of the anti-mE1E2_bv_ VLP antiserum toward DENV serotypes 1 and 2 was more evident when the recombinant E proteins were replaced by the corresponding EDIII proteins, as the coating antigen (**Figure 4B**). The serotype-specific preferential reactivity toward EDIIIs of serotypes 1 and 2 was also displayed by antisera elicited by the pmE1+E2 VLPs (**Figure 4E**). Closer examination of the data using EDIII coating antigens reveals that ELISA titers, specific to DENV serotypes 1 and 2, for both anti-mE1E2_bv_ VLP (**Figure 4B**) and pmE1+E2 VLP (**Figure 4E**) antisera are about an order of magnitude higher than those for DENV serotypes 3 and 4. This is presumably a reflection of efficient display of EDIIIs of serotypes 1 and 2 on the mVLP surface. Using infectious DENVs as the coating antigen, we once again observed that the anti-mE1E2_bv_ VLP antiserum manifested relatively higher ELISA titers against serotypes 1 and 2 (**Figure 4C**), an observation which was once again mirrored by the anti-pmE1+E2 antiserum as well (**Figure 4F**). However, ELISA titers obtained using DENVs as the coating antigen, were roughly one log lower compared to those using the recombinant purified protein coating antigens. This is presumably a result of lower coating density achievable with infectious DENVs as opposed to purified recombinant proteins. These data demonstrate that the mE1E2_bv_ VLPs are highly immunogenic. Further, the immunogenicities of its two components are comparable to their monovalent counterparts.

### mE1E2_bv_ VLPs Elicit EDIII-Focused nAbs Specific to DENV Serotypes 1 and 2

**FIGURE 5:**
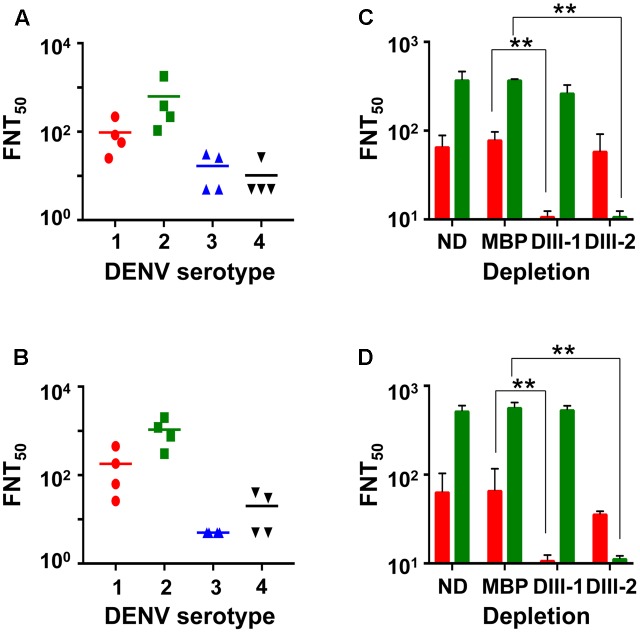
The mE1E2_bv_ VLPs elicit EDIII-specific nAbs to DENV-1 and DENV-2. Virus-neutralizing titers (FNT_50_) of antisera from BALB/c mice immunized with mE1E2_bv_ VLPs **(A)** and pmE1+E2 VLPs **(B)** against DENV-1 (red circles), DENV-2 (green squares), DENV-3 (blue upright triangles), and DENV-4 (black inverted triangles), determined using the FACS based assay. Four independent biological replicate experiments were performed for each DENV serotype. Differences among the data points within each of the four datasets in both **(A,B)** were statistically insignificant. The short horizontal line represents the geometric mean FNT_50_ titer. **(C,D)** represent DENV-1 (red bars) and DENV-2 (green bars) FNT_50_ titers of anti-mE1E2_bv_ VLP antisera and anti-pmE1+E2 VLP antisera, respectively, which were either not subjected to depletion (ND) or depleted with immobilized MBP (MBP), MBP-EDIII-1 (DIII-1), or MBP-EDIII-2 (DIII-2). Values shown are the mean of two biological replicate experiments. The difference between FNT_50_ titers between the ND and MBP datasets against DENV-1 and DENV-2 were insignificant. Statistically very significant differences (*p* < 0.001) are indicated by double asterisks.

Having demonstrated that the mE1E2_bv_ VLPs are immunogenic, we investigated if the antibodies they elicited would be capable of neutralizing infectivity of DENVs. To this end, we evaluated the virus-neutralizing potential of pooled anti-mE1E2_bv_ VLP antiserum against each of the four DENV serotypes in a FACS-based assay, as shown in **Figure 5**. These data are derived from four independent immunizations. Antibodies elicited by the mE1E2_bv_ VLPs efficiently blocked the infectivity of DENV-1 and DENV-2 (**Figure 5A**). However, the antiserum manifested only low level inhibitory potency against DENV-3 and DENV-4. This leads to the conclusion that the mE1E2_bv_ VLPs are capable of eliciting a bivalent nAb response predominantly against DENV-1 and DENV-2. Once again a head-to-head comparison of this bivalent nAb response elicited by the mE1E2_bv_ VLPs was mirrored by the pmE1+E2 VLPs as well (**Figure 5B**), suggesting that E1 and E2 components of the mE1E2_bv_ VLPs are as potent at eliciting nAbs as their cognate counterparts in the physical mixture.

Next, we carried out depletion experiments wherein we pre-treated the anti-mE1E2_bv_ VLP antiserum with recombinant MBP fusions of EDIII-1 or EDIII-2 immobilized on amylose resin to remove antibodies specific to EDIII of the cognate DENV serotype. An indirect ELISA using purified recombinant EDIIIs as coating antigens revealed that depletion using immobilized EDIII-1 and EDIII-2 selectively abrogated ELISA titers specific to EDIII-1 and EDIII-2, respectively (Supplementary Figure S4A). However, we also observed modest heterotypic depletion, particularly the depletion of anti-EDIII-3 antibody titers by immobilized EDIII-1. As expected, control depletion with immobilized MBP did not produce any discernible effect on the indirect ELISA titers. Comparison of the indirect ELISA titers in anti-pmE1+E2 VLP antisera post-depletion revealed essentially similar data (Supplementary Figure S4B).

We also estimated FNT_50_ titers using the FACS-based virus neutralization assay in the anti-mE1E2_bv_ VLP antiserum post-depletion (**Figure 5C**). A control depletion performed with immobilized MBP did not affect virus nAb titer against either DENV-1 or DENV-2. However, depletion with EDIII-1 and EDIII-2, led to significant abrogation of nAb titers against DENV-1 (*p* = 0.0003) and DENV-2 (*p* = 0.0001), respectively. This demonstrates that the nAb response elicited by the mE1E2_bv_ VLPs is predominantly focused on the EDIIIs of E1 and E2 proteins. Once again, these data were mirrored by antiserum from pmE1+E2 VLP-immunized mice (**Figure 5D**).

### Antibodies Elicited by mE1E2_bv_ VLPs Do Not Enhance Sub-lethal DENV-2 S221 Infection *in Vivo*

**FIGURE 6:**
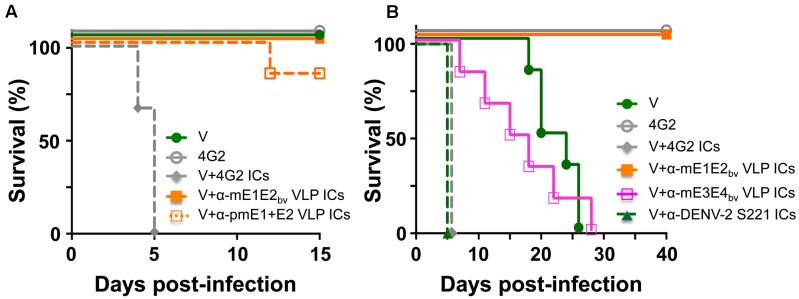
Evaluation of the ADE potential of DENV E-based VLP vaccine-induced antibodies. **(A)** Groups (*n* = 6) of AG129 mice were challenged with a sub-lethal dose of DENV-2 S221 (V) or 100% *in vitro*-neutralized ICs of DENV-2 S221 with mAb 4G2 (V+4G2 ICs), anti-mE1E2_bv_ VLP antiserum (V+α-mE1E2_bv_ VLP ICs) or anti-pmE1+E2 antiserum (V+α-pmE1+E2 VLP ICs) and monitored for survival. One set of mice was inoculated with mAb 4G2 alone (4G2) in the absence of any virus as a survival control. **(B)** Experiment similar to that in **(A)**, performed for extended time duration. Groups (*n* = 6) of AG129 mice were challenged with a sub-lethal dose of DENV-2 S221 (V) or 100% *in vitro*-neutralized ICs of DENV-2 S221 with mAb 4G2 (V+4G2 ICs), anti-mE1E2_bv_ VLP antiserum (V+α-mE1E2_bv_ VLP ICs), anti-mE3E4_bv_ VLP antiserum (V+α-mE3E4_bv_ VLP ICs), or anti-DENV-2 S221 antiserum (V+ α-DENV-2 S221 ICs), obtained from V group in **(A)**. A ‘4G2’ group was included as survival control group as in **(A)**. Survival curves of different groups at 100% have been slightly displaced, to avoid superimposition, to make them visible.

We wished to address the question: would the antibodies in anti-mE1E2_bv_ VLP antiserum enhance DENV infection via the FcR pathway? One approach to this would be to examine the effect of pre-incubating DENV with several dilutions of test antisera/antibodies on its infectivity using a cell line such as K-562 or THP-1 which expresses FcR. However, it is becoming increasingly apparent that such *in vitro* ADE data obtained using FcR-containing cell lines do not mirror the *in vivo* situation (Watanabe et al., 2015; Pantoja et al., 2017). Thus, we turned to an *in vivo* ADE assay to assess the enhancement potential of antibodies elicited by mE1E2_bv_ VLPs. Earlier studies have reported that passive transfer of anti-DENV antisera followed 24 h later with live virus challenge can cause ADE in AG129 mice which lack interferon α/β and γ receptors (Balsitis et al., 2010; Zellweger et al., 2010). This is because ICs formed by the interaction of DENVs with cross-reactive antibodies can infect FcR-bearing cells, such as monocytes and macrophages, and cause disease enhancement. More recently, it was shown that ADE can be recapitulated *in vivo* in AG129 by direct inoculation of *in vitro*-generated ICs consisting of non-lethal amounts of DENV-2 S221 and the cross-reactive DENV mAb 4G2 (Watanabe et al., 2015). We utilized this latter model system to assess the ADE-causing potential of antibodies elicited by mE1E2_bv_ VLPs and compared it with that of the antibodies elicited by pmE1+E2 VLPs. To this end, ICs were generated *in vitro* by pre-incubating non-lethal doses of the DENV-2 challenge strain S221 (2 × 10^4^ FIU) either with anti-mE1E2_bv_ VLP antisera or anti-pmE1+E2 VLP antisera, and inoculating the resultant ICs into separate groups of AG129 mice. We ensured that the DENV-2 S221 virus in these ICs was 100% neutralized using the FACS-based DENV neutralization assay. For comparison, we also inoculated a group with ICs (100% neutralized) generated using mAb 4G2 (10 μg). Control groups received either an equivalent non-lethal dose of DENV-2 S221 alone (group ‘V’) or mAb 4G2 alone (**Figure 6A**). Neither of the control groups manifested any mortality for the entire duration of the experiment, which extended 5 days beyond the experimental duration reported by the earlier investigators (Watanabe et al., 2015). This demonstrated that the dosage of DENV-2 S221 employed in our experiment is truly non-lethal. Consistent with the findings of the earlier study (Watanabe et al., 2015), administration of ICs generated using mAb 4G2 into AG129 resulted in 100% mortality by day 5 post-inoculation. Interestingly, ICs generated using anti-mE1E2_bv_ VLP antiserum did not affect mice survival, suggesting that unlike the cross-reactive mAb 4G2 which can enhance a non-lethal infection to a lethal one, antibodies elicited by the mE1E2_bv_ VLPs did not. When this ADE experiment was performed with anti-pmE1+E2 VLP antiserum to generate the ICs, there was a slight, but statistically insignificant (*p* = 0.317), decrease in survival that became evident on day 12 post-challenge. *In vivo* ADE evaluation could not be extended to the other DENV serotypes due to lack of access to the corresponding challenge strains during this study.

### Heterotypic Bivalent VLP Antisera Cause Modest ADE of DENV-2 S221 Infection

Since both the bivalent VLP antisera used in the above experiment (**Figure 6A**) contained high amounts of DENV-2 specific nAbs and the challenge virus (used to generate the ICs) was also of serotype 2, it is likely that homotypic neutralization overcame any possible infection enhancement. The obvious question this raises is: would there be enhancement if the DENV-2 S221 ICs were to be generated using heterotypic antibodies? To address this question, we designed and created mVLPs by co-expressing the E ectodomains of DENV-3 (E3 protein) and DENV-4 (E4 protein), designated as mE3E4_bv_ VLPs (Supplementary Protocol S1, Supplementary Figure S5, and Supplementary Tables S1, S2). Using an immunization schedule as before, we raised anti-mE3E4_bv_ VLP antiserum in BALB/c mice and incubated it *in vitro* with DENV-2 S221 to generate fully neutralized ICs (V+α-mE3E4_bv_ VLP ICs) and inoculated these into fresh AG129 mice, as shown in **Figure 6B**. As these ICs contained DENV-2 S221 neutralized using cross-reactive heterotypic antibodies, we decided to monitor survival beyond the 15-day period of the previous experiment. We observed that survival of mice in the V+α-mE3E4_bv_ VLP IC group began declining incrementally from day 6 with 100% mortality by day 28, demonstrating that these heterotypic nAbs promote ADE. Mice in the V group, which manifested no mortality until about day 18 post-inoculation, began to succumb relatively rapidly, with none live at day 26. This is likely a result of replication of the input dose of DENV-2 S221 to levels high enough by day 18 to promote progressive mortality in the V group. The difference in the survival curves for these two groups (V *vs* V+α-mE3E4_bv_ VLP IC) was statistically insignificant (*p* = 0.999). In striking contrast, mice inoculated with ICs generated from DENV-2 S221 plus anti-mE1E2_bv_ VLP antibodies (V+α-mE1E2_bv_ VLP IC group) did not manifest any sign of mortality for the entire duration of the experiment, up to day 40. This was statistically very significant when compared to the survival of mice in the V group (*p* = 0.0007). In this experiment, we also included a group, V+α-DENV-2 S221 ICs, wherein the ICs were generated using antiserum from the control (V) group of AG129 mice which received only the non-lethal dose of DENV-2 S221 in the earlier experiment (**Figure 6A**). Surprisingly, mice in this group manifested rapid 100% mortality by day 5 post-inoculation, similar to the V+4G2 group (**Figures 6A,B**). Apparently, virus-induced antibodies possess significant ADE potential. Collectively, the data suggest that EDIII-focused homotypic nAbs elicited by *P. pastoris*-expressed recombinant E-containing VLPs do not possess any significant enhancement potential.

### Lack of ADE by Anti-mE1E2_bv_ VLP Antibodies Correlates with Down-regulation of Pro-inflammatory Cytokines

**FIGURE 7:**
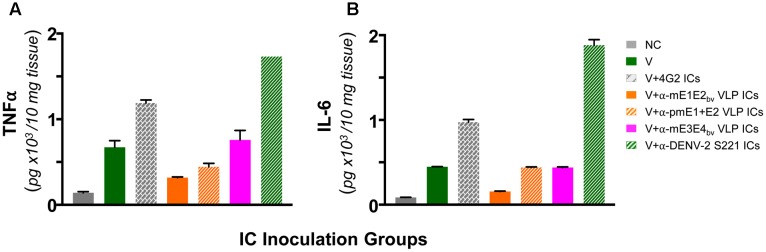
Determination of levels of TNF-α and IL-6 in small intestines of AG129 mice after inoculation of ICs. Groups of AG129 mice (*n* = 3) were challenged with a sub-lethal dose of DENV-2 S221 (V) or 100% *in vitro*-neutralized ICs of DENV-2 S221 with mAb 4G2 (V+4G2 ICs), anti-mE1E2_bv_ VLP antiserum (V+α-mE1E2_bv_ VLP ICs), anti-pmE1+E2 VLP antiserum (V+ α-pmE1+E2 VLP ICs), anti-mE3E4_bv_ VLP antiserum (V+α-mE3E4_bv_ VLP ICs), or anti-DENV-2 S221 antiserum (V+ α-DENV-2 S221 ICs), in parallel with the inoculations described in **Figure 6B**. One group was included as negative control (NC) which did not receive any treatment. Three days post-inoculation mice were euthanized, small intestines dissected out after perfusion and homogenized. Clarified homogenates were used for the determination of TNF-α **(A)** and IL-6 **(B)** using commercial ELISA kits, with purified recombinant murine TNF-α and IL-6 as references. Data shown are the mean values with the bars denoting standard deviation (*n* = 3).

Antibody-dependent enhancement in AG129 mice resulting from inoculation of ICs made using sub-lethal dose of DENV-2 S221 and the cross-reactive mAb 4G2 manifest intestinal pathology. These mice manifest increase in vascular permeability and production of the pro-inflammatory cytokines TNF-α and IL-6 before day 5 following IC inoculation (Watanabe et al., 2015). In other words, these two cytokines can serve as markers to assess the disease status in this model system. This leads to the prediction that these cytokine levels should not be elevated in the small intestines of AG129 mice inoculated with DENV-2 S221 ICs made using anti-mE1E2_bv_ VLP and anti-pmE1+E2 VLP antisera. Data on the levels of TNF-α and IL-6 in small intestines of AG129 mice at 3 days post-inoculation of ICs are presented in **Figure 7**. Basal levels (NC group) of both these cytokines were elevated in mice receiving the sub-lethal dose of DENV-2 S221 (V group). That antibodies elicited by mE1E2_bv_ and pmE1+E2 VLPs did not result in ADE (**Figure 6A**) was corroborated by the finding of lowered levels of small intestinal TNF-α (**Figure 7A**) and IL-6 (**Figure 7B**) in the mice which received ICs generated with anti-mE1E2_bv_ VLP and anti-pmE1+E2 VLP antisera. Reduction in TNF-α levels (**Figure 7A**) in AG129 mice in the ‘V+αE1E2_bv_ VLP ICs’ (*p* = 0.0139) and the ‘V+pmE1+E2 VLP ICs’ (*p* = 0.0453) groups was statistically significant compared to TNF-α levels in the ‘V+4G2 ICs’ group which manifested high mortality due to ADE. Likewise, IL-6 levels in the ‘V+4G2 ICs’ group was significantly higher compared to those in the ‘V+αE1E2_bv_ VLP ICs’ (*p* = 0.0147) and ‘V+pmE1+E2 VLP ICs’ (*p* = 0.0331) groups. In fact the levels of both these cytokines in these groups which do not manifest ADE were closer to basal levels. Anti-pmE1+E2 VLP antibodies appeared relatively less effective at decreasing small intestinal pro-inflammatory cytokine production. Consistent with the previous report (Watanabe et al., 2015), mAb 4G2, which caused vigorous ADE, was associated with elevated levels of both these pro-inflammatory cytokines. Interestingly, the ADE manifested by the heterotypic anti-mE3E4_bv_ VLP antibodies (**Figure 6B**) was mirrored by increased levels of both these cytokines over basal levels, comparable to those observed in the V group of mice. Massive increase in small intestinal TNF-α and IL-6 levels was seen in AG129 mice receiving ICs generated using anti-DENV-2 S221 antiserum (V+α-DENV-2 S221 ICs). It is of note that this group also manifested severe ADE, akin to that mediated by mAb 4G2 in the earlier experiment (**Figure 6B**). Collectively, the cytokine data corroborate the conclusion that antibodies elicited by E1 and E2 containing VLPs do not promote enhancement of sub-lethal DENV-2 S221 infection.

## Discussion

It is becoming increasingly evident that a successful dengue vaccine must not only elicit potent serotype-specific nAbs against all four DENVs, but it must also not manifest any potential for disease enhancement. ADE associated with Dengvaxia (Halstead and Russell, 2016) may be linked to two factors: one, interference among the four replicating vaccine virus strains (Edelman, 2011; Thomas, 2011; Swaminathan et al., 2013), and two, the presence of prM (Dejnirattisai et al., 2010; Rodenhuis-Zybert et al., 2010; Smith et al., 2016) in the vaccine. This makes it imperative to consider non-replicating and prM-lacking dengue vaccine alternatives.

We showed earlier that the DENV-2 E protein ectodomain expressed in *P. pastoris* in the absence of prM self-assembled into VLPs (Mani et al., 2013). More recently, we confirmed that this observation is true for the E protein ectodomains of the remaining three DENV serotypes as well (Tripathi et al., 2015; Poddar et al., 2016; Khetarpal et al., 2017). From a vaccine perspective, these VLPs possess many critically important attributes. Firstly, they possess intrinsically low ADE potential as they lack prM, which can elicit enhancing antibodies (Dejnirattisai et al., 2010; Rodenhuis-Zybert et al., 2010; Smith et al., 2016). Further, the question of viral interference does not arise as they are non-replicating entities. Second, these VLPs display EDIII, a domain of the E protein possessing potent type-specific neutralizing epitopes and involved in host receptor recognition (Lindenbach et al., 2013; Pierson and Diamond, 2013), very efficiently on the surface. Third, these VLPs are highly immunogenic eliciting predominantly homotypic EDIII-focused virus-nAb responses (thus, these are monovalent VLPs). Fourth, these VLPs are produced using *P. pastoris*, a eukaryotic host system with a capacity for high productivity using inexpensive media. Collectively, these attributes have the potential to make the E VLP-based vaccine safe, efficacious, and cost-effective.

The current work is driven by the hypothesis that co-expression of multiple E proteins in a single *P. pastoris* host may allow their co-assembly into mVLPs containing different E molecules. This stems from: (i) our earlier observation that recombinant E molecules of the four DENVs possess inherent self-assembling potential as mentioned above; and (ii) the E molecules share ∼60% homology (Pierson and Diamond, 2013). If indeed such multivalent mVLPs can be generated, they would be more cost-effective compared to multivalent formulation comprising individually prepared monovalent E VLPs. Thus, we explored the feasibility of developing multivalent mVLPs, as an alternate approach to dengue vaccine development. In a first step, we created a bivalent expression clone of *P. pastoris* by integrating *E1* and *E2* ECs in a head-to-tail tandem arrangement into its *AOX1* locus. Next, we showed that it can co-express both the corresponding mRNAs upon methanol-induction. The E1 and E2 proteins were co-purified by Ni^2+^-NTA affinity chromatography and found to co-assemble into mVLPs displaying the EDIIIs of E1 and E2.

Having demonstrated that co-expression of E1 and E2 resulted in bivalent mVLPs (mE1E2_bv_ VLPs), on the basis of a customized sandwich ELISA (designed to detect both E1 and E2 associated with each other), DLS and EM analyses, we next sought to evaluate their immunogenicity in BALB/c mice. In order to assess if the incorporation of E1 and E2 into mE1E2_bv_ VLPs influenced their individual immunogenic potencies, we carried out a head-to-head comparison of these with a physical mixture of monovalent E1 and E2 VLPs (pmE1+E2 VLPs) in all the immunogenicity analyses. The mE1E2_bv_ VLPs were highly immunogenic, eliciting antibodies that recognized all four DENV serotypes, using recombinant Es, EDIIIs or infectious DENVs as the coating antigens in an indirect ELISA format. However, ELISA titers were consistently biased more toward DENV serotypes 1 and 2. This cross-reactivity is the natural consequence of the ∼60% similarity among the DENV serotypes (Pierson and Diamond, 2013). However, in striking contrast we observed that virus nAb titers were predominantly focused toward DENV-1 and DENV-2, but not toward DENV-3 and DENV-4. In fact, EDIII antibody depletion experiments showed that the neutralizing antibody titers are almost entirely directed toward EDIIIs of DENV serotypes 1 and 2. That >90% of the nAb titers are EDIII-focused is evidence that they constitute the predominant DENV neutralizers in the total antibody repertoire induced by these E based VLPs.

Some of the observations made during the analysis of the immune responses elicited by the bivalent mE1E2_bv_ VLPs are intriguing. For example, while ELISA titers were consistently higher toward DENV serotype 1 than toward serotype 2, nAb titers were consistently greater against DENV-2 compared to DENV-1. This could be a reflection of subtle inherent differences in the overall folding and disposition of the *P. pastoris*-expressed E monomers in the VLPs, which presumably allow EDIII of E2 to be displayed more efficiently than EDIII of E1. Apparently, this is at the cost of total ELISA titer against DENV-2, and may explain the observation of higher nAb titers, but lower ELISA titers against DENV-2, with respect to these two parameters for DENV-1.

Interestingly, the anti-EDIII antibodies elicited by E1 did not neutralize DENV-2. Similarly, E2-elicted anti-EDIII antibodies did not neutralize DENV-1. This demonstrated that E1 and E2 in mE1E2_bv_ VLPs elicit exclusively homotypic nAbs, consistent with our earlier observations with the cognate monovalent E VLPs (Mani et al., 2013; Poddar et al., 2016). This is an important finding, as recent work has indicated that serotype-specific nAbs do not manifest ADE in an *in vivo* mouse model (Watanabe et al., 2015). We corroborated this conclusion experimentally by generating ICs of a non-lethal dose of the challenge virus, DENV-2 S221, with antibodies elicited by the mE1E2_bv_ VLPs in BALB/c mice, and inoculating them into AG129 mice. Though this mouse model is susceptible to ADE, it is not considered an ideal host as it lacks a functional interferon signaling pathway, and may not necessarily be reliable in predicting outcome in the human context. Thus, data using this model needs to be interpreted with caution. The anti-mE1E2_bv_ VLP antibodies did not convert the non-lethal infection to a lethal infection whereas the cross-reactive mAb 4G2 (or antiviral antiserum)-generated ICs with the non-lethal dose of the challenge virus killed all the AG129 mice by post-inoculation day 5. That anti-mE1E2_bv_ VLP antibodies did not mediate disease enhancement was also confirmed by cytokine analysis. Increased cytokine production is a characteristic feature of severe dengue disease (Martina et al., 2009). It has been shown in the IC-inoculation ADE model that there is a distinct small intestine pathology marked by elevation in TNF-α and IL-6 production (Watanabe et al., 2015). The levels of these two cytokines in the small intestine of AG129 mice inoculated with ICs containing sub-lethal dose of DENV-2 S221 complexed to antibodies elicited by mE1E2_bv_ VLPs were suppressed to levels below those seen in mice inoculated with an equivalent dose of the virus alone. This leads to the conclusion, with the caveat of the AG129 model’s limitations, that anti-mE1E2_bv_ VLP antibodies may possess less ADE potential.

On the other hand, it could be argued that the higher DENV-2-specific nAb titers in anti-mE1E2_bv_ VLP antisera (**Figure 5**), neutralized the challenge virus (also of the same serotype) efficiently, overcoming any possible enhancement. In other words, our experiment may have been biased against detecting ADE of DENV-2, as it mirrored a homotypic scenario. While experimental evidence is not available for or against this argument, we can offer the following explanation. First, in our ADE experiment, effectively ∼5 μl antiserum (used to generate the ICs) inoculated into an AG129 mouse would undergo significant dilution. While this would result in reduction of DENV-2 specific nAb titers by ∼2 orders of magnitude, we do not observe any ADE in the case of ICs prepared using anti-mE1E2_bv_ VLP-induced antibodies. This in fact is contrary to prevalent thinking that upon sufficient dilution even nAbs can enhance homotypic infection (Pierson et al., 2007). Second, the observed nAb titers for DENV-1 and DENV-2 in our studies may not be very different from each other, in comparison to nAb titers of ∼10, considered as evidence of effective seroconversion in vaccine trials. It is to be pointed out that the most appropriate means to test if our experimental system was biased in favor of not detecting ADE of DENV-2, would be to evaluate ADE of DENV-1. At the present time this is not feasible due to lack of access to a challenge DENV-1 strain. However, the observation that we are able to score ADE of DENV-2 using heterotypic anti-mE3E4_bv_ VLP-induced antibodies (see below) shows that our assay evaluates ADE reliably.

Another interesting finding was the observation of vigorous ADE and high levels of pro-inflammatory cytokines in the small intestines of AG129 mice inoculated with sub-lethal doses of DENV-2 S221 complexed to antibodies in anti-DENV-2 S221 antiserum. This antiserum was found to possess high nAb titers (FNT_50_ ∼4,000). This is a seemingly enigmatic finding given that nAb titers were high and it represents a homotypic scenario. While the reasons for this are unclear, it is likely that cross-reactive antibodies, especially anti-prM antibodies in the anti-DENV-2 antiserum, are responsible for ADE. It has been shown that inoculation of highly neutralized ICs is accompanied by infusion of large amounts of free antibodies (Watanabe et al., 2015). Many of these antiviral antibodies, being cross-reactive could potentially generate more infectious ICs *in vivo* by complexing with newly generated DENV-2, resulting in more severe ADE. This is reflected by the observation that mice in this experiment were the ones that displayed the highest levels of small intestinal pro-inflammatory cytokine production.

Importantly, in all the immunogenicity evaluation studies, the mE1E2_bv_ VLPs behaved very similarly to the pmE1+E2 VLP mixture, suggesting that the bivalent VLPs retain the inherent immunogenic potential of its monovalent constituents intact. These conclusions were by and large also true for mE3E4_bv_ VLPs and the antibodies elicited by them, with the exception that, anti-mE3E4_bv_ VLP antibodies promoted ADE of DENV-2. This is consistent with these heterotypic antibodies lacking any neutralizing efficacy against DENV-2. The ADE caused by these heterotypic antibodies was associated with elevated cytokine levels over basal levels, especially for TNF-α.

## Conclusion

Our work demonstrates the feasibility of creating multivalent mVLPs, representing at least two DENV serotypes. The bivalent VLPs not only preserved the serotype-specific antigenic identity of their monovalent precursors, but also displayed the cognate EDIIIs on their surface eliciting potent, EDIII-focused nAb responses to the two precursor serotypes. Though humans produce only low levels of anti-EDIII antibodies in response to DENV infection, our results suggest the feasibility of re-focusing the immune response to EDIII and thereby facilitating the induction of a robust EDIII-directed nAb response. Further, the EDIII-focused, exclusively homotypic nature of nAb response is important in the light of recent work which has indicated that serotype-specific nAbs do not manifest ADE in an *in vivo* mouse model (Watanabe et al., 2015). That the predominantly EDIII-focused antibodies elicited by the mE1E2_bv_ VLPs did not enhance a sub-lethal dose of DENV-2 S221 is consistent with this notion. The lack of ADE correlated with suppression of pro-inflammatory cytokines TNF-α and IL-6. Based on this work, we hypothesize that tetravalent mVLPs containing E1, E2, E3, and E4, may offer a single 4-in-1 vaccine candidate with the capacity to not only elicit protective EDIII-focused neutralizing response targeting all four DENV serotypes but may also be free of ADE potential and may provide a lead for a future safe, efficacious and cost-effective vaccine alternative.

## Author Contributions

Cloning, expression, purification, physical and immunological characterization of mE1E2_bv_ VLPs: RS. Similar work relating to mE3E4_bv_ VLPs: RR. PCR, RT-qPCR, DLS, and EM analyses: RS and RR. Antigen formulation and mouse immunizations: UA. Virus neutralization assays: AP. Study design and conception: SS and NK. Analysis and interpretation of data: RS, RR, UA, SS, and NK. Drafting of the manuscript: SS and NK. Approval of final manuscript: RS, RR, UA, AP, SS, and NK.

## Conflict of Interest Statement

The authors declare that the research was conducted in the absence of any commercial or financial relationships that could be construed as a potential conflict of interest.
