# Supplementary material for: Pichia pastoris-Expressed Bivalent Virus-Like Particulate Vaccine Induces Domain III-Focused Bivalent Neutralizing Antibodies without Antibody-Dependent Enhancement in Vivo
Source: Front Microbiol. 2018 Jan 9;8:2644. doi: 10.3389/fmicb.2017.02644 (PMC5768101; doi:10.3389/fmicb.2017.02644)
Supplement: Supplementary file 1 [file Data_Sheet_1.pdf]

Pichia pastoris-expressed bivalent virus-like particulate vaccine induces domain III-focused bivalent neutralizing antibodies without antibody-dependent enhancement *in vivo*

Supplementary Material

Protocol S1

Production and characterization of mE3E4$_{bv}$ VLPs: In order to co-express the E proteins of DENV-3 and DENV-4, a strategy similar to that employed for the co-expression of E proteins of DENV-1 and DENV-2 was employed (Figure S5). We first created monovalent DENV-3 E (E3) and DENV-4 E (E4) ECs, using codon-optimized synthetic *E* genes encoding the C-terminal 34 *aa* residues of prM protein, followed by the first 395 *aa* residues of the E protein of the cognate DENV serotype and a 6x His tag**,** in the plasmid pAO815. The *AOX1* promoter-driven E3 and E4 ECs were assembled in a head-to-tail manner to create the bivalent construct pDENV-E3E4$_{bv}$ (Figure S5A). This was integrated into *P. pastoris* GS115 (*his4*) genome to obtain a bivalent clone harboring E3 and E4 ECs *via* selection for His^{+}/*Mut*^{S} transformants. Genomic DNA of this E3E4-bivalent clone was subjected to PCR analysis using E3 and E4 gene-specific primers (Table S1) to confirm successful integration of both ECs into the host genome (Figure S5B). The expression of E3- and E4-encoding mRNAs was verified through RT-qPCR (Figure S5C) and the co-expressed proteins co-purified using Ni^{2+}-NTA affinity chromatography. The capacity of the co-purified E3 and E4 proteins to co-aggregate into higher order structures was assessed by DLS (Figure S5D). Integrity of the EDIII epitopes of the E3 and E4 proteins was corroborated by indirect ELISA using a battery of type-specific murine conformational mAbs (Table S2). Collectively, these data showed that the E3 and E4 proteins, co-expressed in *P. pastoris* and co-purified, co-assembled into mosaic VLPs, which were designated as mE3E4$_{bv}$ VLPs. The immunogenicity of these VLPs was evaluated in BALB/c mice (Figure S5E).

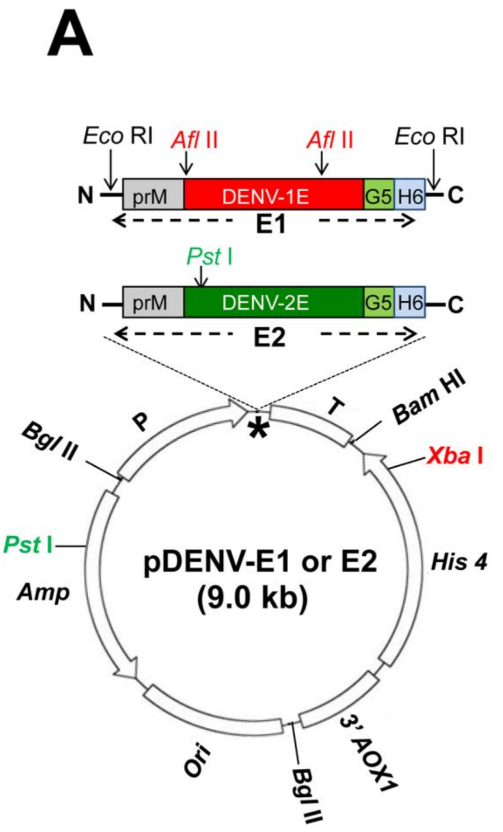

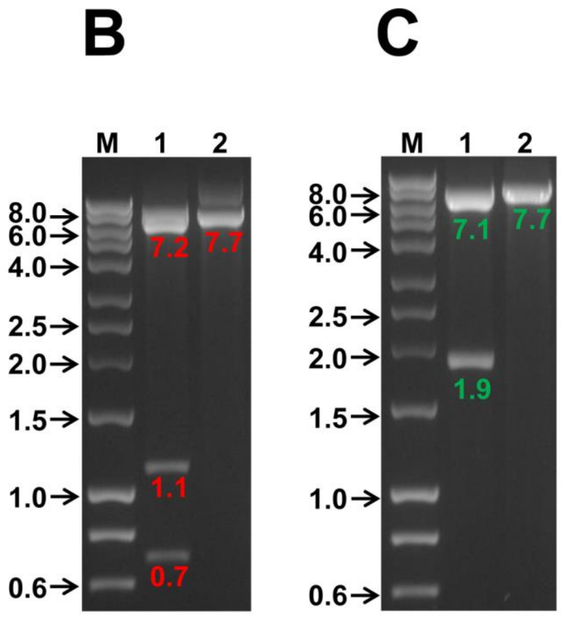

Insert orientation	pDENV-E1	pDENV-E2
	Afl II & *Xba* I digestion	*Pst* I digestion
Correct (kb)	7.2, 1.1, 0.7	7.1, 1.9
Incorrect (kb)	7.8, 0.7, 0.5	6.0, 3.0

Figure S1: Construction of monovalent DENV E-expressing vectors, pDENV-E1 and pDENV-E2. (A) Map of the yeast integrative vector pAO815 containing either the DENV-1 *E* gene (E1, shown in red) or the DENV-2 *E* gene (E2, shown in green) inserted into the unique *Eco* RI site (indicated by the asterisk). Each of the two DENV *E* genes (encoding the ectodomain spanning *aa* residues 1-395) is flanked by sequences encoding the C-terminal 34 *aa* residues of prM (gray box) on the 5' side and by sequences encoding penta-glycine linker (G5, shown in light green) plus a stretch of six-histidine residues (H6 shown in light blue) on the 3' side. In these plasmids the DENV *E* gene is placed under the transcriptional control of the *AOX1* promoter (P) on the 5' side and the *AOX1* transcriptional terminator (T) on the 3' side. These plasmids contain the *HIS4* marker (histidinol dehydrogenase) for selection in the *P. pastoris* strain GS115 (*his 4)* and sequences from the 3' end of the *AOX1* gene (*3' AOX1*), required for genomic integration into *P. pastoris*. They also possess the bacterial origin (*Ori*) and ampicillin (*Amp*) resistance markers, respectively, for propagation and selection in *E. coli*. Restriction sites in the vector and insert used for identifying the pDENV-E1 and pDENV-E2 plasmids are shown in red and green fonts, respectively. (B) Restriction analysis using *Afl* II and *Xba* I to verify correct orientation of insert in pDENV-E1. (C) Restriction analysis using *Pst* I to verify correct orientation of insert in pDENV-E2. In panels 'B' and 'C', the restriction digests of the recombinant plasmid and the parent vector were analyzed in lanes 1 and 2, respectively. Band sizes (in kb) are indicated below the bands. DNA size markers were analyzed in lanes 'M'. Their sizes (in kb) are shown on the left of the panels. The table below panels 'B' and 'C' indicates the predicted band pattern for the recombinant plasmids containing the inserts in correct and incorrect orientations.

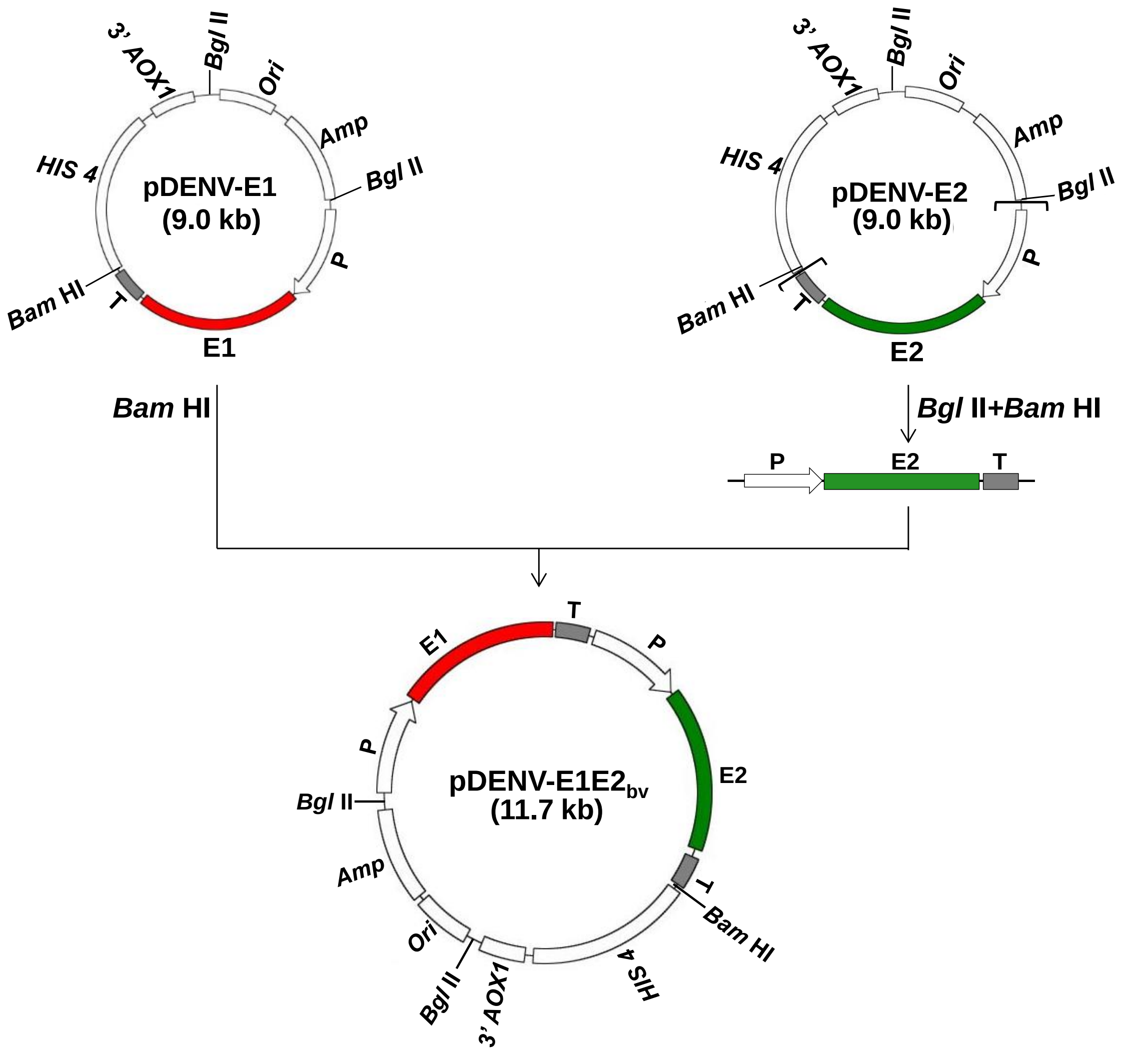

Figure S2: Strategy for the construction of E1 and E2 bivalent expression plasmid pDENV-E1E2$_{bv}$. The bivalent construct was created by excising the *E2* EC from pDENV-E2 as a *Bgl* II-*Bam* HI fragment and inserting it into the unique *Bam* HI site of pDENV-E1. The *E1* and *E2* genes are shown in red and green, respectively, with each carrying nucleotide sequences encoding the C-terminal 34 *aa* residues of the cognate *prM* gene and 6x-His tag at its 5' and 3' ends, respectively. Each *E* gene possesses its own *AOX1* promoter (P) and transcription terminator (T) sequences. The bivalent plasmid contains the *HIS4* marker (histidinol dehydrogenase) for selection in the *P. pastoris* strain GS115 (*his 4)* and *3' AOX1* sequences, required for genomic integration into *P. pastoris*, similar to the starting monovalent plasmids. Like its monovalent precursors, the bivalent plasmid also possesses the bacterial origin (*Ori*) and ampicillin (*Amp*) resistance markers, respectively, for propagation and selection in *E. coli*.

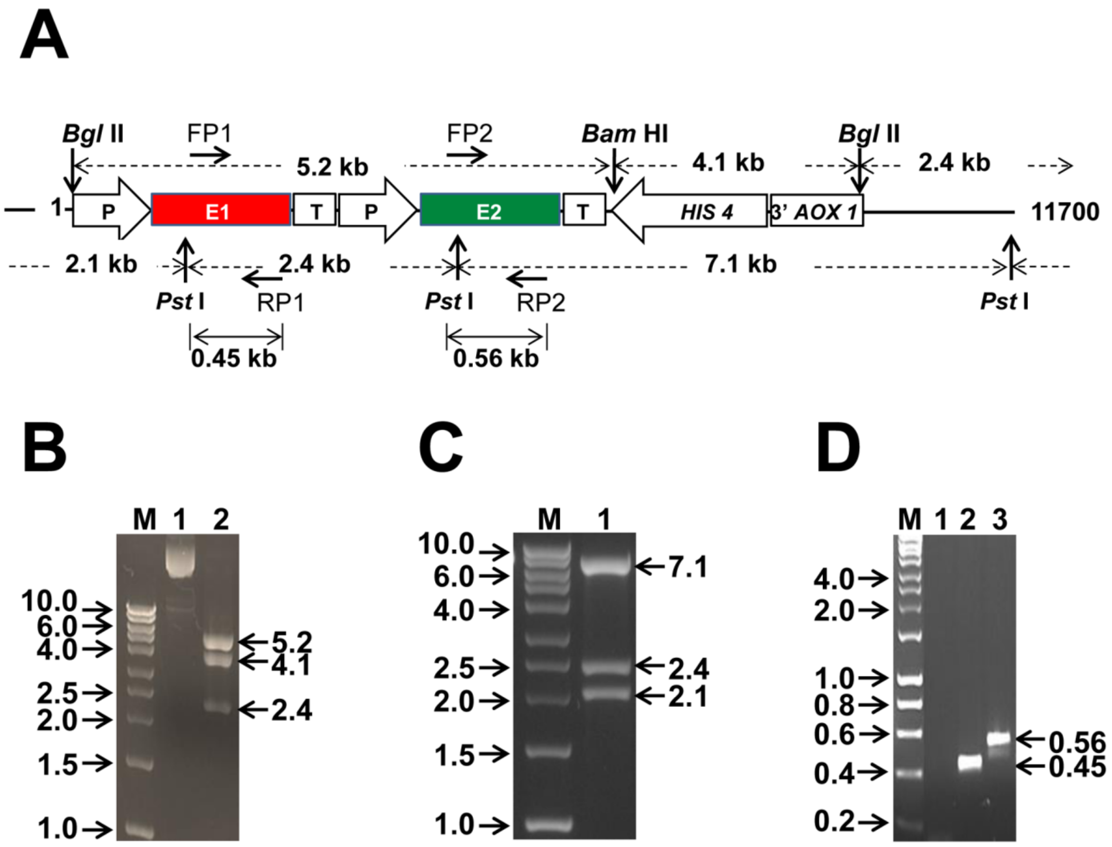

Figure S3: Physical characterization of the bivalent pDENV-E1E2$_{bv}$ construct. (A) Schematic representation of the tandem DENV-1 and DENV-2 *E* gene ECs. The restriction sites and the distances separating them (dashed double-headed arrows), PCR primer-binding sites (horizontal rightward and leftward solid arrows) and predicted amplicons (solid double-headed arrows) are indicated. All sizes are indicated in kb. (B) Agarose gel analysis of pDENV-E1E2$_{bv}$ construct, before (lane 1) or after (lane 2), digestion with *Bam* HI+*Bgl* II. (C) Agarose gel analysis of pDENV-E1E2$_{bv}$ construct after digestion with *Pst* I (lane 1). (D) PCR analysis of genomic DNA extracted from *P. pastoris* after integration of the tandem expression cassettes and *HIS4* marker, using *E1*-specific (FP1 and RP1, lane 2) and *E2*-specific (FP2 and RP2, lane 3) primers, indicated in panel 'A'. PCR primer sequences are indicated in Table S1. A control PCR reaction (with no template) was analyzed in lane 1. DNA size markers were analyzed in lanes 'M' of panels B-D. Their sizes (in kb) are shown on the left of each panel. Shown to the right are the predicted restriction fragment (panels 'B' and 'C') and PCR product (panel 'D') sizes (in kb).

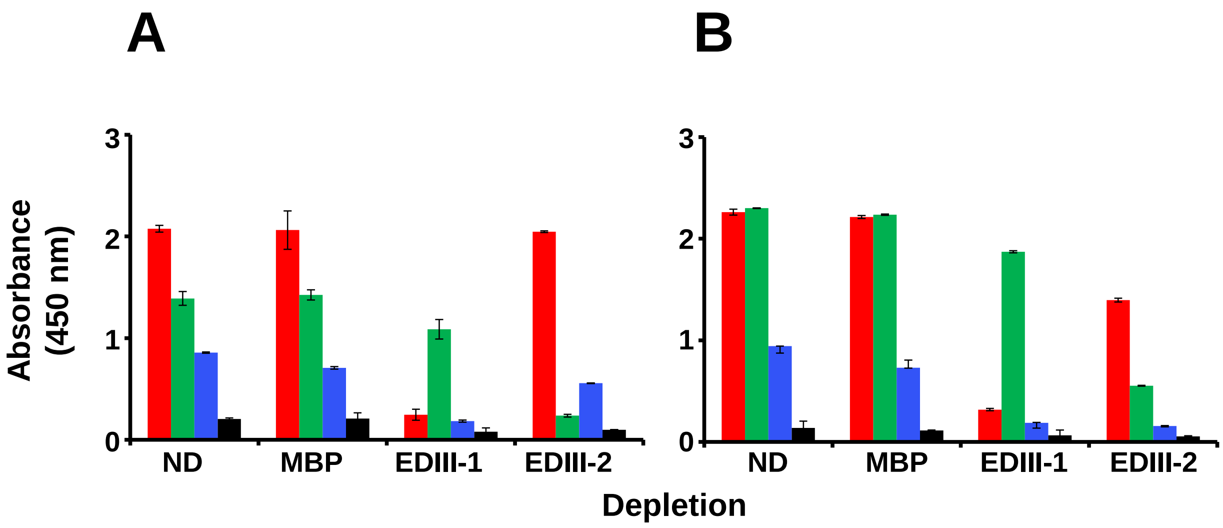

Figure S4: Analysis of EDIII-specific antibody titers by indirect ELISA. (A) Anti-mE1E2$_{bv}$ VLP antisera and (B) anti-pmE1+E2 VLP antisera which were either not subjected to depletion (ND) or depletion on immobilized MBP (MBP), EDIII-1-MBP (EDIII-1) or EDIII-2-MBP (EDIII-2), were analyzed for residual anti-EDIII antibody titers using purified recombinant EDIII-1 (red bars), EDIII-2 (green bars), EDIII-3 (blue bars) or EDIII-4 (black bars) as the coating antigen (all 4 EDIII proteins were in the form of MBP fusions). Data shown are the average of duplicates. The vertical line segments indicated SD.

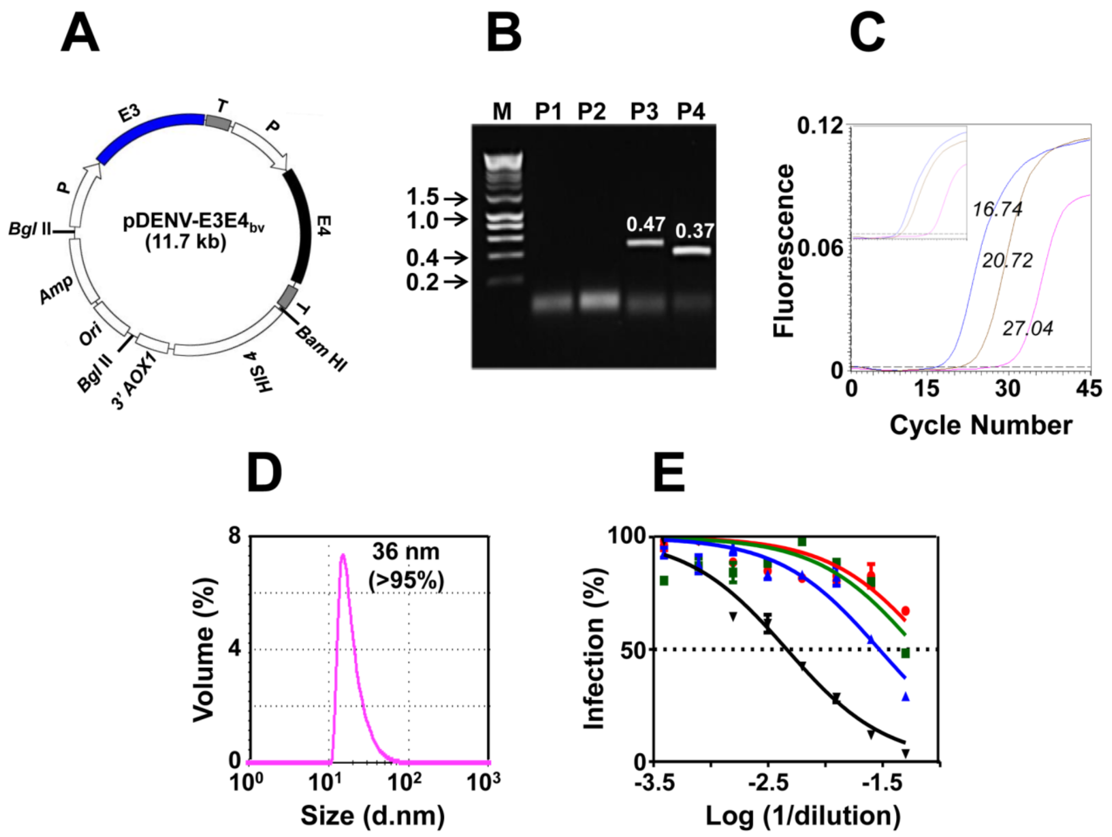

Figure S5: Design and characterization of mE3E4$_{bv}$ VLPs. (A) Map of the pDENV-E3E4$_{bv}$ plasmid. The plasmid contains two tandem ECs with one encoding DENV-3 E (E3, shown in blue), and the other DENV-4 E (E4, shown in black), each with its own *AOX1* promoter (P) and transcription terminator (T) sequences. Both *E3* and *E4* genes carry nucleotide sequences encoding the C-terminal 34 *aa* residues of the cognate prM genes and 6x-His at their 5' and 3' ends, respectively. Present on the 3' side of the E4 EC is the *HIS4* marker for selection in the *P. pastoris* strain GS115 (*his4)*, followed by *3' AOX1* sequences, required for genomic integration. The plasmid also carries the bacterial origin (*Ori*) and ampicillin (*Amp*) resistance markers, respectively, for its propagation and selection in *E. coli*. (B) Genomic PCR analysis of *P. pastoris* clone harboring *E3* and *E4* ECs of the plasmid in panel 'A'. Genomic DNA was analyzed by PCR using primer pairs (see Table S1), specific to the *E* genes of DENV-1 (P1), DENV-2 (P2), DENV-3 (P3) and DENV-4 (P4), followed by agarose gel electrophoresis. DNA size markers were analyzed in lane 'M'. Their sizes (kb) are shown on the left. The sizes (kb) of the expected amplicons are shown above the bands. (C) Real time analysis of DENV *E3* and *E4* mRNA expression in *P. pastoris* transformed with pDENV-E3E4$_{bv}$. Total RNA was isolated from the methanol-induced bivalent *P. pastoris* clone, transcribed to cDNA using reverse primers specific to either DENV-3 *E* (blue) or DENV-4 *E* (brown) and subjected to qPCR. Total RNA from *P. pastoris* transformed with the empty expression vector pAO815 was subjected to RT-qPCR in parallel as a negative control (pink). The C_t values are shown for each amplification profile. The inset (same axes scales as for the main figure) shows the RT-qPCR amplification profiles obtained using total RNA extracted from *P. pastoris* transformed with pDENV-E3 (blue), pDENV-E4 (brown) or pAO815 (pink). (D) DLS analysis of purified mE3E4$_{bv}$VLPs. The average diameter of the VLPs is shown at the top of the peak; the value in parenthesis indicates the proportion of these VLPs in the purified preparation. (E) Virus-neutralizing activity of antisera from BALB/c mice immunized with mE3E4$_{bv}$ VLPs against DENV-1 (red curve), DENV-2 (green curve), DENV-3 (blue curve) and DENV-4 (black curve), as a function of serum dilution, was determined using the FACS based assay. The dotted horizontal line represents 50% infection.

Table S1: Primers for genomic PCR analysis of *P. pastoris* clones harboring DENV *E* genes.

**Primer pair**[a]	**Primer Name**	**Sequence (5'-3')**	**Length**	**Amplicon size (bp)**
1	FP1	GGATACTAACTTTGTCTGTAGAAGAACATTCGTT	34	452
	RP1	GCAAGTCCTGTCTATTCCAGGTTTCTTGACTTGTTGAA	38	
2	FP2	AATCAAGATTACTCCACAGTCTTCCATTACCGAAGCA GAG	40	562
	RP2	AAGAACGTGTCTCTTCTCCAAGTCCATAATTTCG	34	
3	FP3	GATCTTAGCCTTATTTCTTGCCCACTATATC	31	474
	RP3	TTCGATGGACTCCAAGCATTGA	22	
4	FP4	GGTCGATTCCGGTGACGGAAACCATA	26	373
	RP4	TCTGAACCAGTGCAAAGTAAGAGCGGAATTTCCAA	35	

[a]Primer pairs 1, 2, 3 and 4 were designed to be specific to the E genes of DENV-1, -2, -3 and -4, respectively.

Table S2: Analysis of E-specific epitope integrity of the mE3E4$_{bv}$ VLPs[a]

**mAb**[b]	**Serotype specificity**	**Epitope Specificity**	**ELISA OD$_{450nm}$**				
			**E3E4$_{bv}$**	**E1**	**E2**	**E3**	**E4**
E12	DENV-1	EDIII	0.12	1.82	0.07	0.12	0.07
E24		EDIII	0.08	1.23	0.03	0.02	0.03
E27		EDIII	0.08	1.25	0.02	0.02	0.02
E29		EDIII	0.20	1.87	0.03	0.09	0.14
3H5	DENV-2	EDIII LR	0.03	0.03	1.84	0.06	0.04
E1	DENV-3	EDIII	3.33	0.04	0.03	3.43	0.02
E3		EDI/II	2.17	0.13	0.03	2.90	0.02
E4		EDI/II	2.59	0.12	0.04	3.18	0.03
E29	DENV-4	EDIII	1.92	0.04	0.04	0.03	2.80
E42		E	1.17	0.03	0.02	0.02	2.10
E43		E	2.17	0.02	0.02	0.02	2.15

[a]The analysis was carried out by indirect ELISA using mE3E4$_{bv}$ VLPs as the coating antigen; the DENV serotype specificity of the mAbs was analyzed in parallel using *P. pastoris*-produced monovalent E protein VLPs corresponding to DENV-1 (E1), DENV-2 (E2), DENV-3 (E3) and DENV-4 (E4). Yellow highlighted cells indicate significant ELISA reactivity. Data shown are the averages of duplicates.

[b]mAbs E12, E24, E27 & E29: Shrestha *et al*, 2010; mAb 3H5: Henchal *et al*, 1982; mAbs E1, E3 & E4: Brien *et al*, 2010; mAbs E29, E42 & E43: Sukupolvi-Petty *et al*, 2013.
